# SPINK1-driven cellular heterogeneity and interaction networks in intrahepatic cholangiocarcinoma revealed by integrated multi-omics analysis

**DOI:** 10.1016/j.jare.2025.11.011

**Published:** 2025-11-17

**Authors:** Xiaoxiang Rong, Jiayun Guan, Chanqi Ye, Qiong Li, Mingyu Wan, Wenguang Fu, Jinzhang Chen, Dayong Zheng, Ruyin Chen, Rui Zhou, Gautam Sethi, Yunlu Jia, Jian Ruan

**Affiliations:** a Department of Oncology, Nanfang Hospital, Southern Medical University, Guangzhou 510515, China; b Department of Medical Oncology, the First Affiliated Hospital, Zhejiang University School of Medicine, Hangzhou 310003, China; c Department of Pharmacology and NUS Centre for Cancer Research (N2CR), Yong Loo Lin School of Medicine, National University of Singapore, 117600, Singapore; d Department of Hepatobiliary Surgery, The Affiliated Hospital of Southwest Medical University, Luzhou 646000, Sichuan Province, People's Republic of China; e Department of Infectious Diseases, Nanfang Hospital, Guangdong Provincial Key Laboratory for Prevention and Control of Major Liver Diseases, Southern Medical University, Guangzhou, China; f Department of Oncology, Shunde Hospital, Southern Medical University, The First People's Hospital of Shunde, Foshan, China

**Keywords:** SPINK1, Intrahepatic cholangiocarcinoma, Tumor microenvironment, Metabolic reprogramming, Immunosuppression

## Abstract

**Introduction:**

Intrahepatic cholangiocarcinoma (ICC) is a highly heterogeneous and aggressive malignancy with poor prognosis and limited treatment options. The lack of precise molecular classification and incomplete understanding of the tumor microenvironment (TME) impede therapeutic development.

**Objectives:**

This study aims to dissect the cellular heterogeneity and intercellular interactions in ICC, with a particular focus on SPINK1-overexpressing epithelial subsets. We sought to determine the role of SPINK1 in modulating immune cell recruitment and tumor metabolism, and to evaluate its clinical relevance.

**Methods:**

We performed single-cell RNA sequencing (scRNA-seq) on human ICC tumors and adjacent normal tissues to construct a transcriptomic atlas. Integrative proteomic analysis, transwell assays, co-culture systems, and functional perturbation experiments (SPINK1 knockdown and ASCT2 inhibition) were conducted to explore epithelial–macrophage interactions and metabolic dynamics.

**Results:**

ScRNA-seq identified a SPINK1-overexpressing epithelial subcluster enriched in ICC tumors. High SPINK1 expression correlated with significantly shorter patient survival. SPINK1-overexpressing epithelial subcluster secretes CCL20, which recruits lipid-associated macrophages (LAMs). This effect was reversed by CCL20 neutralizing antibodies. In co-culture, LAMs increased intracellular glutamine levels in SPINK1-overexpressing epithelial subcluster, promoting proliferation. Disruption of SPINK1 expression or glutamine transport abolished this metabolic support.

**Conclusion:**

Our findings uncover a novel SPINK1–CCL20–LAMs axis that orchestrates immune recruitment and metabolic reprogramming in ICC. SPINK1 facilitates tumor growth by establishing a glutamine-rich microenvironment via LAMs. These insights highlight SPINK1 as a potential therapeutic target and prognostic biomarker in ICC.

## Highlight

• SPINK1-Expressing Tumor Cells Recruit LAMs via CCL20 to Drive Immunosuppression and Metabolic Reprogramming.
• Silencing SPINK1 suppresses tumor growth, metastasis, and glutamine uptake in vitro and in vivo, highlighting its therapeutic potential.
• Multi-Omic Integration Reveals E4_SPINK1-CAF Crosstalk in Reshaping the TME.
• Pan-Cancer Significance of SPINK1 in Tumor Immunity and Metabolism.

## Graphical abstract

An integrative multi-omics analysis of intrahepatic cholangiocarcinoma (ICC) reveals a highly malignant epithelial subpopulation (E4_SPINK1) characterized by SPINK1 overexpression. E4_SPINK1 cells secrete the chemokine CCL20 to recruit lipid-associated macrophages (LAMs), which in turn synthesize glutamine via elevated GLUL activity. The released glutamine is imported into E4_SPINK1 cells through SLC1A5, fueling tumor proliferation, invasion, and immunosuppression. Concurrently, E4_SPINK1 engages matrix cancer-associated fibroblasts (matrix_CAFs) via FAM3C–HLA-C and SPP1–CD44 ligand–receptor axes, driving extracellular matrix remodeling. Therapeutically, SPINK1 knockdown disrupts CCL20-mediated LAM recruitment, reduces glutamine uptake, and impairs tumor growth and metastasis. Pan-cancer analyses further identify SPINK1 as a prognostic biomarker and regulator of tumor immunity and metabolism.

**Figure:**
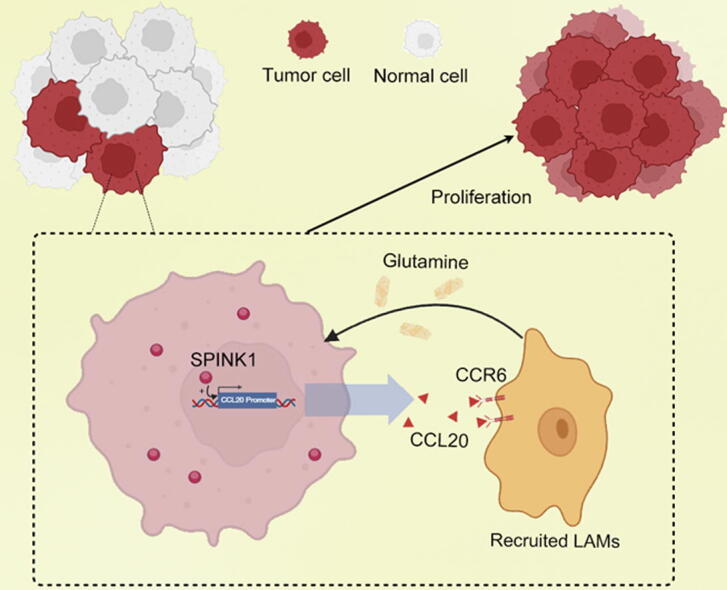

## Introduction

Intrahepatic cholangiocarcinoma (ICC) is the second most common primary liver cancer, accounting for approximately 20 % of all hepatic malignancies [1,2]. Despite advancements in therapeutic approaches, the 5-year overall survival rate remains low, at only 9 % [3]. Furthermore, the incidence of ICC continues to rise [4]. Currently, surgical resection is considered the optimal treatment option, but only 20–30 % of patients are eligible for surgery [5]. Even after successful surgical intervention, the prognosis for ICC patients remains poor [6]. The adverse prognosis of ICC can largely be attributed to the complexity and continuous evolution of its tumor microenvironment (TME), particularly the pronounced desmoplastic stromal reaction [7]. Studies have demonstrated that the TME plays a pivotal role in promoting disease progression, therapeutic resistance, and immune suppression in ICC [[8], [9], [10]]. Therefore, elucidating the dynamic changes in the TME during ICC development is crucial for identifying potential therapeutic targets and improving patient outcomes.

TME constitutes a complex ecosystem composed of various coexisting and interacting components, including tumor-associated macrophages (TAMs), cancer-associated fibroblasts (CAFs), dendritic cells, and others [11]. Through dynamic interactions with these TME constituents, tumor cells play a pivotal role in tumor initiation, progression, metastasis, and drug resistance [12]. For instance, myofibroblastic CAFs (myCAFs) promote intrahepatic cholangiocarcinoma (ICC) development by expressing hyaluronan synthase 2, whereas inflammatory CAFs (iCAFs) enhance ICC growth through the secretion of hepatocyte growth factor [13]. In recent years, metabolic reprogramming within the TME has emerged as a hallmark of malignancy [14]. This metabolic shift supports the rapid proliferation and invasive capacity of cancer cells, thereby significantly driving tumor progression [15,16]. However, research on the specific mechanisms underlying TME metabolic reprogramming in ICC remains limited. Whether metabolic reprogramming contributes critically to the initiation and progression of ICC remains inconclusive. Given the central role of the TME in the poor prognosis of ICC, investigating the interplay between ICC and TME metabolic reprogramming is of paramount importance, not only for elucidating the molecular mechanisms of the disease but also for the development of novel therapeutic strategies.

SPINK1 (Serine Peptidase Inhibitor Kazal Type 1) is a serine protease inhibitor primarily known for its ability to inhibit enzymes such as trypsin [17]. It is highly expressed in the pancreas, where it protects tissues from autodigestion and is associated with hereditary pancreatitis [18]. Recent studies have shown that SPINK1 is overexpressed in various types of cancers, including prostate cancer [19], pancreatic cancer [20], and hepatocellular carcinoma [21]. This overexpression may influence tumor progression by modulating protease activity, thereby affecting the TME [22]. In the context of cancer, SPINK1 has been implicated in mediating chemoresistance through its interactions with the TME [23]. Specifically, SPINK1 can regulate the secretion of cytokines such as IL-6 and TGF-β [24,25], which promote the polarization of macrophages towards an M2 phenotype [26]. This shift enhances immune suppression by inhibiting T cell activity and facilitating immune escape mechanisms within the TME [27]. Additionally, SPINK1′s role in regulating matrix metalloproteinases (MMPs) adds another layer of complexity to its impact on cancer progression [28]. Thus, SPINK1 appears to play a multifaceted role in cancer biology by influencing protease activity, modulating immune responses, and impacting ECM dynamics [29]. These diverse functions highlight the importance of further investigating SPINK1′s mechanisms of action within the TME to better understand its contributions to tumor progression and identify potential therapeutic targets.

In our study, we employed an integrative multi-omics framework, incorporating single-cell genomics, proteomics, and spatial transcriptomics, to dissect the molecular underpinnings of ICC. Our analyses revealed a robust association between elevated expression of SPINK1 and unfavorable prognosis in ICC patients. This multifaceted approach further elucidated a complex interaction network driving ICC progression, centered on the crosstalk between SPINK1-overexpressing tumor cells and LAMs within TME. Specifically, we found that SPINK1 overexpression enhances ICC malignancy by promoting tumor cell proliferation and invasiveness while simultaneously orchestrating the recruitment of LAMs through the upregulation of CCL20, a chemokine known to mediate immune cell trafficking. In response to this signaling, LAMs exhibit a metabolically active phenotype characterized by heightened lipid metabolism, which in turn sustains ICC growth by providing a supportive microenvironmental niche. These findings underscore the pivotal role of SPINK1 in reshaping the TME to favor tumor progression.

## Methods

A comprehensive description of the experimental design, animal grouping criteria, and detailed protocols for scRNA-seq analysis are available in the Supplementary Methods section.

### BD Rhapsody single-cell sequencing

RNA isolation and cDNA library construction for the six samples (comprising matched pairs of tumor (n = 3) and adjacent normal tissues (n = 3) from three ICC patients) were performed following BD Biosciences’ standard procedures (Hangzhou, China). A limiting dilution strategy captured over 10,000 cells. Oligonucleotide-barcoded beads paired with cells in microwells, and after cell lysis, polyadenylated RNA molecules hybridized to the beads. Reverse transcription was carried out, and cDNA was tagged with unique cell-specific barcodes. scRNA-seq libraries were constructed using the BD Rhapsody platform and sequenced on an Illumina Novaseq 6000 (Hangzhou, China). Alignment of FASTQ files to the GRCh38 human reference genome was conducted using STAR software (version 2.7.4a) with default settings [30]. Gene-barcode matrices were generated by counting unique molecular identifiers (UMIs) and filtering non-cell-associated barcodes, producing gene-barcoded matrices for each sample.

### scRNA-seq data processing and quality control

The matrix of read counts per gene per sample was further analyzed by the Seurat package (version 4.0.2) in the R software (version 4.1.3) [31]. For each cell, we used five quality control (QC) measures. Cells meeting any of the following criteria were excluded: (1) cells expresses less than 500 genes or more than 7200 genes [31]; (2) cells contains number of UMIs more than 35,000 [32]; (3) log10GenesPerUMI＜0.8; (4) cells with more than 20 % mitochondrial reads; (5) cells express more than 2 % RBC genes.

### Across–sample integration

The gene expression matrix was normalized using the “NormalizeData” function. To avoid batch effects between samples, the single-cell data were integrated using the canonical correlation analysis (CCA) ensemble method of “Seurat” [33]. As in a previous study [33], 3000 variable features for CCA (canonical correlation analysis) [34] were chosen based on stabilized variance, and integration anchors were identified using the first 20 reduced dimensions.

### Dimension reduction and unsupervised clustering

Principal components analysis (PCA) was performed on the integration-transformed expression matrix using the “RunPCA” function, and the first 50 principal components (PCs) were used in the “FindNeighbors” function. The “FindClusters” function was used for cell clustering, and different parameters which depends on cluster tree were applied to different cell types, 0.6 for all cells, 0.7 for epithelial cells, 0.9 for myeloid cells, 0.9 for fibroblasts (Fig. S1). Dimensionality reduction was performed using the “RunTSNE” function and visualized using T-distributed Stochastic Neighbor Embedding (t-SNE).

### Cell cluster annotation, malignant cell identification, and GSVA analysis

We applied the “FindAllMarkers” function in Seurat to identify the marker genes of each cell cluster/subset, and we manually annotated the cell cluster/subset based on the marker genes. And after extracting specific cell subsets, we used the same method for more detailed annotation. To identify malignant cells from the epithelium, we used “inferCNV” (version 1.18.1) [35]. Neutrophils and B cells were used as normal references to estimate copy number variations (CNVs). When performing CNV scoring, we compared the CNV cumulative score with the reference cells, and cells with CNV scores close to those of normal cells were considered non-malignant, while others were considered malignant. Differential expression analysis between malignant and non-malignant cells was performed using the “FindAllMarkers” function with the parameters set to “min.pct = 0.25, logfc.threshold = 0.75, adjusted p value < 0.01”. GSVA was performed using the “GSVA” package.

[36]. The datasets “C2: curated gene sets” and “C5: ontology gene sets” were downloaded from the Gene Set Enrichment Analysis (GSEA) website (https://www.gsea-msigdb.org/gsea/index.jsp).

### Cellular fraction calculation and correlation with public datasets

For each cell subset, we calculated its infiltration score in ICC tissues and adjacent tissues. Subsequently, we used the bulk RNA sequencing datasets from The Cancer Genome Atlas (TCGA) and Gene Expression Omnibus database (GEO) to predict the relative abundance of each cell subset using the CIBERSORTx algorithm (https://cibersortx.stanford.edu/) [37].

### Tandem mass tag-based proteomic analysis

Formalin-fixed paraffin-embedded (FFPE) tumor samples from the ICC patients cohort (n = 75) were dewaxed with xylene and rehydrated. Tissues were then subjected to acidic hydrolysis with formic acid (FA; Thermo Fisher Scientific). Proteins were denatured with 6 M urea (Sigma–Aldrich) and 2 M thiourea (Sigma–Aldrich) with the assistance of pressure-cycling technology (PCT; Pressure BioSciences Inc; 30 s 45,000 psi and 10 s ambient pressure, 90 cycles). Proteins were then digested into peptides with trypsin (1:20; Hualishi) and Lys-C (1:80; Hualishi) with the assistance of PCT (50 s 20,000 psi and 10 s ambient pressure, 120 cycles). The detailed settings have been described previously [38,39]. The resulting peptides were labeled using the TMTpro 16-plex kit (Thermo Fisher Scientific). Each batch contained 15 experimental samples and one pooled sample in the TMT126 channel for normalization. The labeled peptides were fractionated using offline high-pH reversed-phase fractionation on a Thermo Dionex Ultimate 3000 RSLCnano System (Thermo Fisher Scientific). The fractions were then combined and further separated on the same LC system equipped with a reversed-phase C18 column (1.9 μm, 120 Å, 150 mm × 75 μm i.d.). The LC mobile phases consisted of buffer A (2 % acetonitrile, 0.1 % FA) and buffer B (98 % acetonitrile, 0.1 % FA). Peptides were eluted using a 60-minute linear gradient from 5 % to 28 % buffer B at a flow rate of 300 nL/min. The eluted peptides were analyzed online using a Q Exactive HF mass spectrometer (Thermo Fisher Scientific) operated in positive ion mode with data-dependent acquisition (DDA). The raw mass spectrometry data were processed using Proteome Discoverer (version 2.4, Thermo Fisher Scientific) and searched against the UniProt human database. The detailed parameters have been described previously without modification [40,41]. Among the 10,888 proteins identified, 6,311 proteins were detected in samples with less than 10 % missing values and were subjected to further analysis.

### Survival analysis

RNA sequencing data of paired ICC tissues and adjacent tissues as well as clinical data were obtained from the TCGA and GEO databases to evaluate the prognostic role of gene sets derived from specific cell states. We used the “surv_cutpoint” function of the “survminer” package to determine the optimal cutoff point [42]. This method has been validated across multiple independent cohorts, demonstrating the robustness of this cutoff value. Survival curves were plotted using the Kaplan-Meier method of the Survival package (v.3.2.11) and visualized using the “ggsurvplot” function of the “survminer” package. Significance was assessed by the log-rank test statistic (p-value) between the two groups (patients were stratified into SPINK1 high-expression group and SPINK1 low-expression group based on the expression level of SPINK1).

### Multiplex immunofluorescence (mIF) of ICC tissue

Resected tumor tissues from ICC patients were fixed with 4 % paraformaldehyde before embedding in paraffin. First, the prepared tissue sections (4 mm) were baked in an oven at 65 ℃ for 1 h, dewaxed with xylene, and rehydrated with graded alcohol. After rehydration, the sections were fixed in 10 % neutral buffered formalin (Solarbio, G2161) at room temperature for 20 min, placed in an appropriate AR buffer (AKOYA Biosciences, NEL820001KT), and then placed in a microwave for 1 min at 100 % power and then for an additional 15 min at 20 % power. The slides were blocked with Blocking/Ab Diluent (AKOYA Biosciences, NEL820001KT) after the slides cooled at room temperature and incubated with primary antibody at room temperature for 10 min. TBST (Solarbio, T1082) was used to wash the slides three times, and then the slides were incubated with Opal Polymer HRP MsþRb (AKOYA Biosciences, NEL820001KT) to cover tissue sections at room temperature for 10 min, generally 100 to 300 mL per slide. The slides were rinsed with TBST three times before incubation with Opal Signal Generation (AKOYA Biosciences, NEL820001KT). The microwave treatment, blocking, primary antibody incubation, introduction of Opal Polymer HRP, and signal amplification were repeated. Primary antibodies in this assay including anti-CD68 (Abcam, clone EPR23917-164, 1:200), anti-SPP1 (Abcam, clone RM1018, 1:500), anti-α-SMA (Abcam, clone EPR18430, 1:300), anti-Periostin (Abcam, clone RM1074, 1:100), anti-SPINK1 (Abcam, clone EPR17585-116, 1:100), and anti-panCK (Abcam, C-11, 1:300). Once six targets were labeled, DAPI working solution (AKOYA Biosciences, NEL820001KT) was applied in the dark for 5 min at room temperature, and the slides were washed with distilled water and TBST before mounting. Finally, a confocal microscope (Nikon) was used to take images of those tissue samples, and the acquired images were analyzed using ImageJ (version 4.0), positive/negative expression was used for staining thresholds.

### Mammary orthotopic tumor xenograft assays

Six-week-old female BALB/c Nude mice (n = 5 per group) were used for orthotopic implantation. Control shRNA or SPINK1 shRNA cells (1.0 × 10⁶) were resuspended in 50 μl of 50 % Matrigel and injected into the right thoracic mammary fat pads of each mouse. After 4 weeks, mice were sacrificed, and primary tumors were excised for measurement of weight and volume. All mice were housed in a specific pathogen-free facility (Zhejiang University Animal Care Services) with ventilated cages and sterilized food and water. All animal experiments were approved by the Institutional Animal Care and Use Committee of Zhejiang University (Approval number: 2024292). All surgery was performed under ether anesthesia, and all efforts were made to minimize suffering.

### Establishment a Murine model of intrahepatic cholangiocarcinoma lung metastases

To establish an ICC lung metastasis mouse model, we cultivated ICC cells transfected with a SPINK1 shRNA or control shRNA cells plasmid in vitro until they exhibited vigorous growth. Cells were resuspended in PBS at a concentration of 1 × 10^7 cells/mL. A volume of 200 μL of the cell suspension, containing 2 × 10^4 cells per mouse, was injected into the tail veins of SCID mice. Mice were monitored for several weeks to observe signs of adverse reactions or disease progression. Upon the appearance of significant adverse reactions, mice were euthanized humanely. Dissections were performed to examine the lungs for visible metastases. Lung tissues were cleaned, photographed, fixed in 10 % formalin, and processed into paraffin-embedded sections. H&E staining and IHC staining were conducted. To assess metastatic efficiency, three distinct areas were randomly selected from the tissue sections, and the number of positive cells out of every 100 cells was counted.

### Statistics analysis

Data are presented as the mean ± standard error of the mean (SEM) from at least three independent experiments. The normality of data distribution was assessed using the Shapiro–Wilk test. For comparisons between two groups, the unpaired two-tailed Student’s *t*-test was used for normally distributed data; otherwise, the Mann–Whitney *U* test was applied. For comparisons among more than two groups, one-way ANOVA followed by Tukey’s post hoc test was used for parametric data, and the Kruskal–Wallis test followed by Dunn’s post hoc test was used for non-parametric data. A p-value < 0.05 was considered statistically significant. All analyses were performed using GraphPad Prism 7.0.

## Result

### Single-cell transcriptome profiling and cell phenotyping of intrahepatic cholangiocarcinoma.

**Fig. 1:**
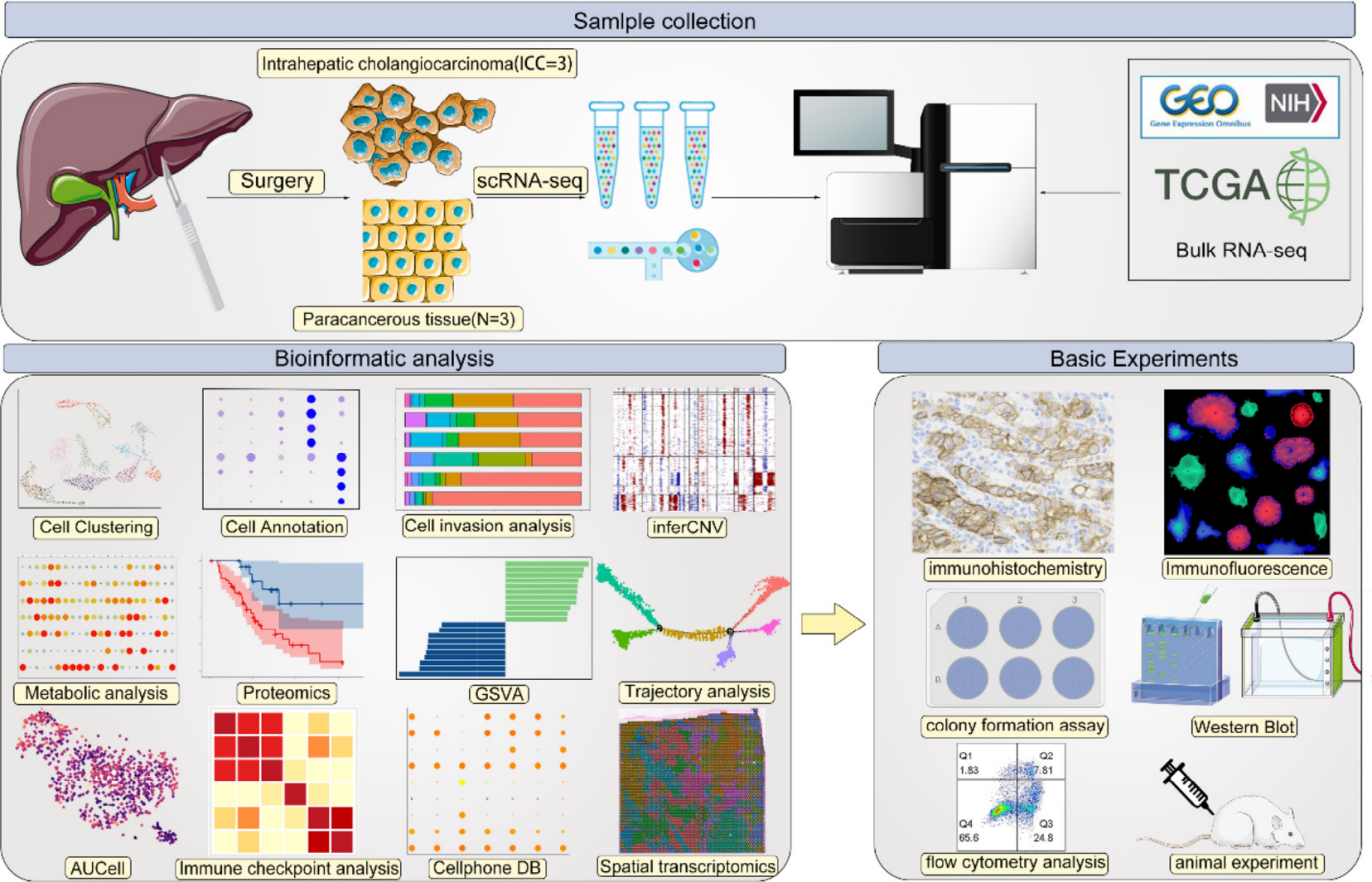
Workflow of sample collection, data analysis and experiments in this study.

**Fig. 2:**
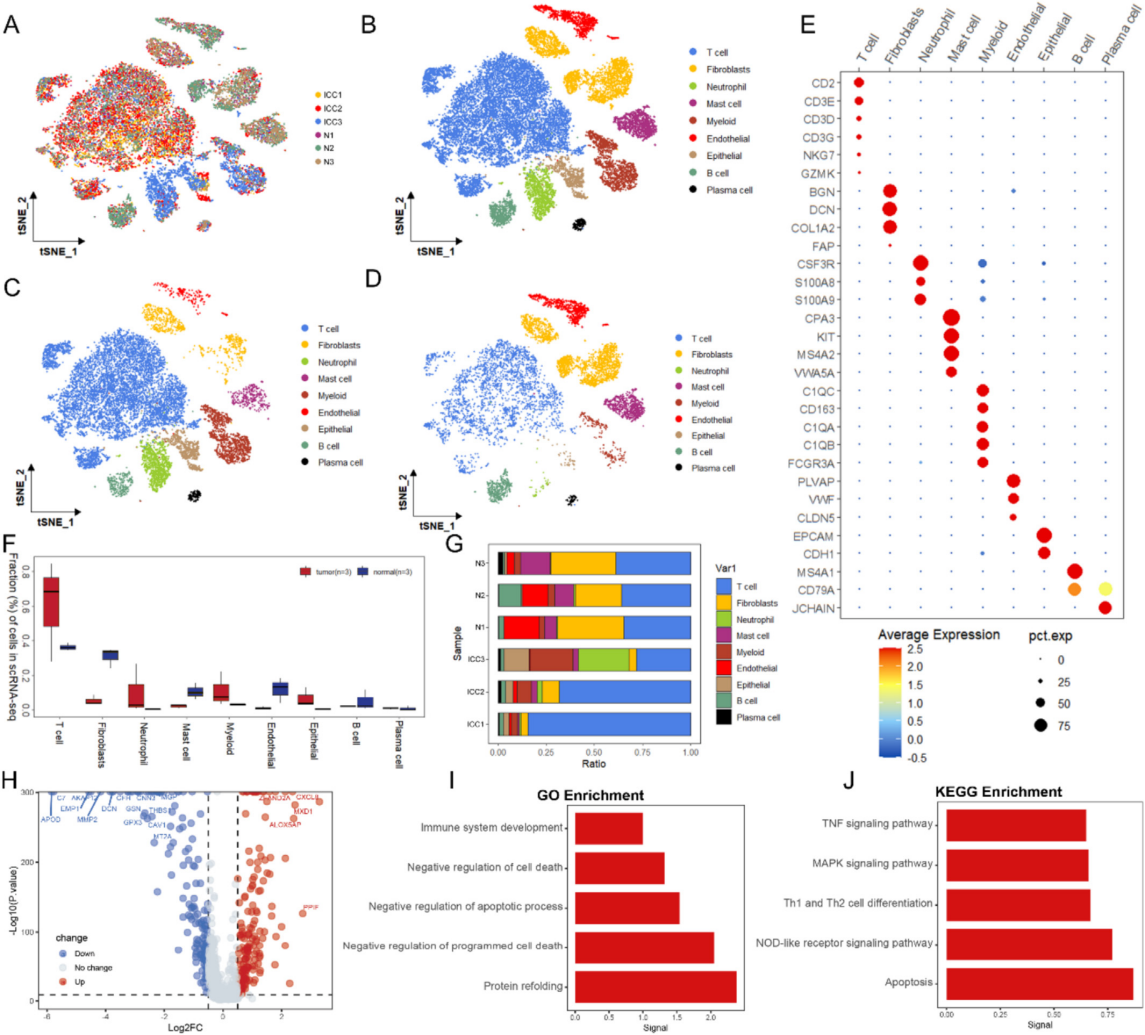
Single-cell transcriptomic analysis in ICC and tumor adjacent tissues. A. T-distributed stochastic neighbor embedding (tSNE) plot shows batch effects between different samples. B. tSNE plot displaying the integrated cell map, which consists of 9 annotated cell types. C-D. tSNE plot showing the integrated cell map of ICC tissue(C) and tumor adjacent tissue(D). E. Dot plot showing representative marker genes across cell clusters. Dot size is proportional to the fraction of cells expressing specific genes. Color intensity corresponds to the relative expression of specific genes. F. Bar plot showing the cell type abundance for samples from different tissue, as measured by scRNA-seq data in this study. G. Bar plot indicating the proportion of major cell lineages in each patient. H. The volcano plot illustrates the differential gene expression between tumor tissue and normal tissue. Genes represented in red indicate upregulation in tumor tissue relative to normal tissue, while those in blue indicate downregulation. I-J. The bar plots display the results of GO (I) and KEGG (J) enrichment analyses for the tumor tissue.

To investigate the TME in ICC, fresh tissue samples were collected post-surgery from three ICC patients, including three tumor samples and paired adjacent normal tissues. Our research workflow is illustrated in Fig. 1. scRNA-seq was performed using the BD Rhapsody platform. After applying rigorous quality control filters—excluding damaged cells, dead cells, and doublets—we retained a total of 22,245 high-quality cell transcriptomes for downstream analysis. This dataset comprised 14,139 cells derived from tumor tissues and 8,106 cells from adjacent normal tissues. To mitigate batch effects and facilitate integrated analysis across tumor and normal samples, we employed Canonical Correlation Analysis (CCA), as illustrated in Fig. 2A. After eliminating batch effects using the CCA approach, we observed that the overall cellular populations of ICC tissues and normal tissues were uniformly intermingled, indicating the successful removal of batch effects. In contrast, when batch correction was performed using the Harmony and Scanorama methods, ICC and normal tissue cells were distinctly segregated, suggesting the presence of pronounced batch effects (Fig. S2). For consistency, we independently processed and integrated data from tumor and adjacent samples, followed by cell-type annotation. This integrative approach confirmed the presence of consistent cell populations across both tissue types, with comparable infiltration patterns observed (Figs. 2B–D). Based on established cell-specific markers (Supplementary Table 2), we identified nine predominant cell types: T cells, fibroblasts, neutrophils, mast cells, myeloid cells, endothelial cells, epithelial cells, B cells, and plasma cells (Fig. 2E and Fig. S2B).

To characterize the TME composition, we quantified the relative abundance of these cell types in tumor versus adjacent tissues (Fig. 2F). Our analysis revealed a marked increase in the infiltration of T cells, neutrophils, myeloid cells, and epithelial cells within tumor tissues compared to their normal counterparts. While cell-type proportions remained broadly consistent among patients with the same tissue type (with the exception of patient ICC3), significant heterogeneity was observed between tumor and adjacent tissues (Fig. 2G). This variability likely reflects the distinct developmental trajectories and microenvironmental adaptations associated with ICC progression.

Further insights were gained through differential gene expression analysis between tumor and normal tissues. We identified a significant upregulation of genes linked to cancer progression, such as CXCL8 [43], in tumor tissues, alongside a pronounced downregulation of genes associated with immune activation, including C7 (Fig. 2H). To elucidate the functional implications of these expression changes, we performed GO and KEGG enrichment analyses on the tumor-derived transcriptomes. These analyses highlighted enrichment in pathways such as “Negative Regulation of Programmed Cell Death,” “Negative Regulation of Apoptotic Process,” and “Th1 and Th2 Cell Differentiation” (Fig. 2I–J). These findings suggest a complex interplay between ICC tumor cells and the TME, potentially involving mechanisms that suppress immune-mediated cell death and modulate T-cell differentiation to favor tumor persistence.

### Comprehensive analysis of epithelial cell heterogeneity in ICC and its role in tumor progression and prognosis

**Fig. 3:**
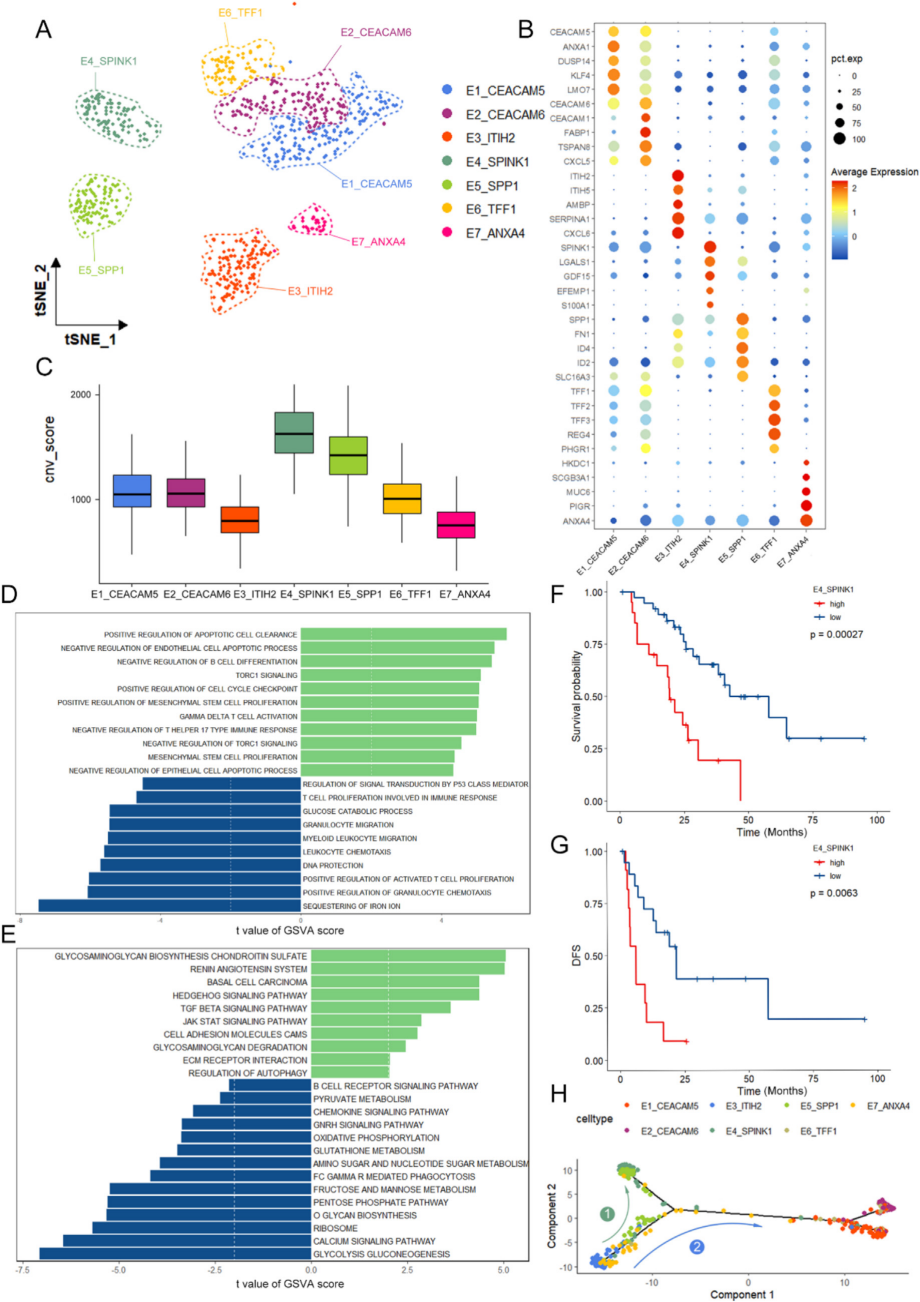
Transcriptional signatures and CNV heterogeneity of Epithelial cells. A. tSNE plot of epithelial cells colored by clusters. B. Dot plot showing representative marker genes of epithelial cells. C. Box plot showing the cnv scores of different clusters. D-E. The GSVA of malignant cells and non-malignant cells using the data sets “C5: ontology gene sets”(D) and “C2: curated gene sets”(E). F-G. Kaplan–Meier curves of GEO&TCGA ICC patients (n = 58) showing the Overall survival(F) and Disease-free survival(G) grouped by the cell abundance in E4_SPINK1. The p value is calculated with two-sided log-rank test. H. Semisupervised pseudotime trajectory of epithelial cells subtypes inferred by Monocle2.

To dissect the heterogeneity within the epithelial cell population of ICC, we identified seven distinct clusters based on specific molecular markers: E1_CEACAM5, E2_FABP1, E3_ITIH2, E4_SPINK1, E5_SPP1, E6_TFF3, and E7_ANXA4 (Fig. 3A–B). Using inferCNV analysis to assess copy number variations (CNVs), we distinguished malignant from non-malignant epithelial clusters. Clusters E3_ITIH2 and E7_ANXA4 were classified as non-malignant, while the remaining clusters exhibited malignant characteristics, with E4_SPINK1 demonstrating the highest CNV scores, indicative of pronounced malignancy (Fig. 3C, Fig. S3A). Notably, CNV scores were significantly elevated in tumor tissues compared to adjacent normal tissues (Fig. S3B). To characterize the functional profiles of these clusters, we conducted Gene Set Variation Analysis (GSVA). Malignant clusters were enriched in biological processes such as positive regulation of apoptotic cell clearance, cell cycle checkpoint regulation, and mesenchymal stem cell proliferation, whereas non-malignant clusters showed enrichment in processes including iron ion sequestration, positive regulation of activated T-cell proliferation, and leukocyte chemotaxis (Fig. 3D). Pathway enrichment analysis further revealed that malignant clusters were associated with the Hedgehog signaling pathway, TGF-β signaling pathway, JAK-STAT signaling pathway, and extracellular matrix (ECM) receptor interactions (Fig. 3E).

We next quantified the distribution of these epithelial subsets across tumor and adjacent tissues. As anticipated, malignant epithelial clusters predominated in tumor tissues, while non-malignant clusters, except for E3_ITIH2, were more prevalent in adjacent normal tissues (Fig. S3C). To validate these observations, we integrated two external RNA-seq datasets: the GEO dataset (GSE107943), comprising 27 paired ICC and normal tissue samples and 3 single ICC samples, and the TCGA dataset, including 7 paired samples, 25 single ICC samples, and 1 single normal sample. After correcting for batch effects, we combined these into a unified GT dataset (n = 97) and applied CIBERSORTx deconvolution to confirm the infiltration patterns of epithelial subsets (Fig. S3C). Survival analysis using a subset of the GT dataset (n = 58) demonstrated that high enrichment of E1_CEACAM5, E2_FABP1, and E4_SPINK1 correlated with poor overall survival, with E4_SPINK1 exhibiting the strongest prognostic significance (Fig. 3F, S4A).

Patients with elevated enrichment of E1_CEACAM5, E2_FABP1, and E4_SPINK1 exhibited a higher propensity for early relapse, whereas those with greater E3_ITIH2 enrichment experienced extended DFS (Fig. 3G, S4B). These findings reinforce the critical role of E4_SPINK1 in ICC initiation, progression, and prognosis. Additionally, E4_SPINK1 enrichment was significantly higher in advanced-stage ICC, while E3_ITIH2 predominated in early-stage disease (Fig. S5A). This stage-specific distribution may be linked to E4_SPINK1′s involvement in suppressing chemokine production, maintaining epithelial structure, and inhibiting IL-8 expression (Fig. S5B).

To elucidate the evolutionary dynamics of epithelial cells during ICC progression, we constructed developmental trajectories using Monocle2. Unsupervised trajectory analysis identified E3_ITIH2 and E7_ANXA4 as the initial states, diverging into two distinct differentiation pathways. Pathway 1, terminating in E4_SPINK1 and E5_SPP1, exhibited the highest CNV scores and was strongly associated with malignant transformation and ICC progression. In contrast, Pathway 2 led to E1_CEACAM5, E2_FABP1, and E6_TFF3 (Fig. 3H). These results suggest that Pathway 1 predominantly drives ICC’s malignant evolution. Furthermore, scMetabolism analysis revealed a metabolic shift in ICC tissues, with a preference for lipid metabolism compared to the amino acid and sugar metabolism dominant in normal tissues (Fig. S6A). This metabolic reprogramming may represent a key driver of ICC pathogenesis, supporting tumor cell survival and proliferation in the TME.

### Clinical significance and functional validation of SPINK1′s role in ICC progression

In light of the pivotal role played TME components in driving ICC progression, it is critical to identify specific molecular markers contributing to these processes. Among these, SPINK1 has been recognized as a key factor in various cancers, including pancreatic cancer [44] and prostate cancer [45]. To validate the presence of E4_SPINK1, we analyzed a publicly available single-cell RNA sequencing dataset (GSE125449) (Fig. S6B). This analysis supported our hypothesis that the SPINK1 gene could be implicated in ICC progression. To validate the prognostic significance of SPINK1 at the protein level, we analyzed its expression in a proteomic dataset comprising 75 ICC samples. Kaplan-Meier analysis confirmed that high SPINK1 protein expression was significantly associated with poor overall survival and increased recurrence risk (Fig. S6C).

**Fig. 4:**
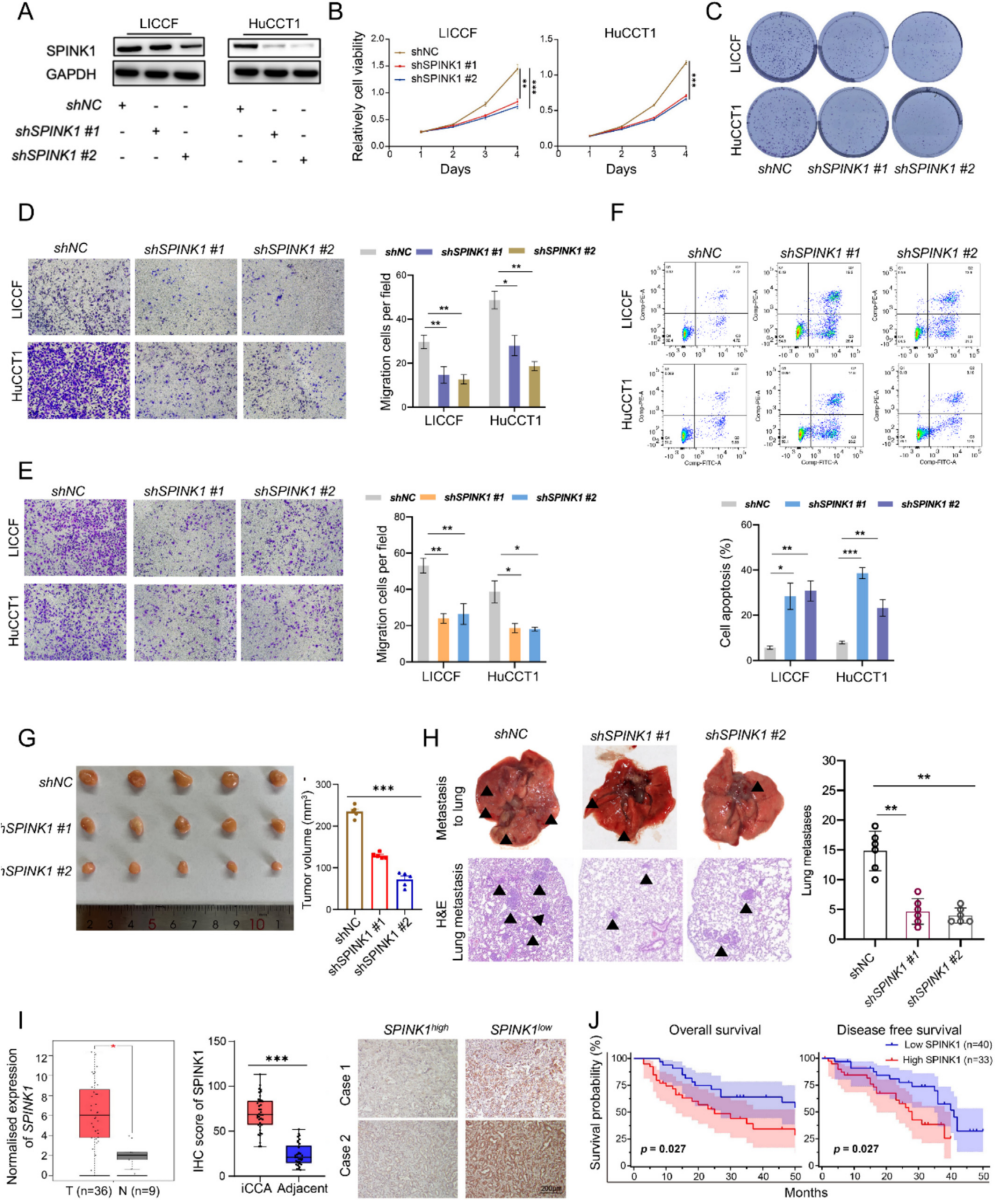
SPINK1 knock-down impaired ICC cell proliferation and migration, which correlated with ICC patient survival. A. Expression levels of SPINK1 protein were detected upon SPINK1 shRNA transfection or the control vector in LICCF and HuCCT1 cells. B. Proliferation of LICCF and HuCCT1 cells were significantly inhibited upon SPINK1 silencing. The cell proliferation was tested every 24 h using CCK8 assays. C. Representative images of colony formation assays of the LICCF and HuCCT1 cell lines. SPINK1 silencing decreased the colony-forming efficiencies in both cell lines. D, E. The cell migration (D) and invasion (E) abilities were measured after transfection with shSPINK1 or shScr in LICCF and HuCCT1 cells. Cells migrating and invading the lower Transwell chambers were counted (magnification, ×200). F. LICCF and HuCCT1 cells were transfected with the CTRL or SPINK1 shRNA vector for 72 h. Examination for apoptotic was performed by annexin V staining and flow cytometry. The percentage of apoptotic cells was represented in a bar diagram from three independent experiments. G. Representative images showing the growth kinetics of LICCF –engrafted tumors in nude mice following SPINK1 knockdown (shSPINK1) compared to the control group. Tumor growth was monitored for four weeks. Quantification of tumor volumes at the endpoint of the experiment. The tumor volume was calculated using the following equation: V = (width2 × length)/2. H. Representative images of lung metastases in mice following tail vein injection of LICCF cells following SPINK1 silencing, then compared to the control group (Left). Quantification of the number of lung metastatic nodules in the HJURP overexpression (Right). I. Analysis of ICC and normal adjacent tissue from the TCGA and surgical samples to determine the expression of HJURP. J. The Kaplan-Meier survival curves and survival analysis presented significant differences in overall survival rates between ICC patients with high and low expressions of SPINK1 (*p* = 0.027). Upon further examination of disease-specific survival, it was observed that ICC patients with high expressions of SPINK1 exhibited significantly lower survival rates compared to those with low expressions (*p* = 0.021).

To investigate the functional impact of SPINK1 on ICC, we selected two human cholangiocarcinoma cell lines, LICCF and HuCCT1, and silenced SPINK1 expression using shRNA plasmids (Fig. 4A). CCK-8 assays revealed a significant reduction in cell viability following SPINK1 knockdown (Fig. 4B). Similarly, colony formation assays demonstrated that silencing SPINK1 markedly inhibited ICC cell proliferation (Fig. 4C). Transwell assays further indicated that SPINK1 knockdown significantly impaired the migration and invasion capacities of ICC cells (Fig. 4D–E). Flow cytometry analysis showed a notable increase in apoptosis among SPINK1-silenced ICC cells compared to controls (Fig. 4F). To extend these findings in vivo, we conducted xenograft studies and established an ICC lung metastasis model using severe combined immunodeficient (SCID) mice. These experiments confirmed that SPINK1 silencing substantially reduced tumor growth and the number of lung metastatic nodules (Fig. 4G–H).

To complement these experimental findings, we analyzed SPINK1 protein expression in clinical samples. IHC staining was performed on 36 ICC tissues and 9 tumor-adjacent normal tissues collected from our hospital. The results revealed significantly higher SPINK1 expression in ICC tissues compared to adjacent tissues (Fig. 4I). To further substantiate these observations, we assembled a clinical cohort of 73 ICC patients. Kaplan-Meier survival analysis demonstrated that patients with high SPINK1 expression exhibited significantly shorter overall survival (OS) and disease-free survival (DFS) compared to those with low SPINK1 expression (Fig. 4J). Additionally, SPINK1 expression was markedly elevated in patients with advanced-stage ICC relative to those with early-stage disease (Fig. S6D). Together, these in vitro, in vivo, and clinical data establish a robust association between elevated SPINK1 expression and the occurrence, progression, and poor prognosis of ICC.

### Prognostic value of SPINK1 and its relationship with tumor immunity across multiple cancer types

To further elucidate the clinical implications of E4_SPINK1 and its representative genetic signatures, and to advance their translational potential into clinical practice, we applied machine learning to identify differentially expressed genes between E4_SPINK1 and other epithelial cell clusters. Using the Gradient Boosting Machine (GBM) algorithm, which exhibited the highest predictive accuracy, we constructed a prognostic model (Fig. S6E), which demonstrated robust predictive efficiency in both the GEO and TCGA datasets (Fig. S6F).

In addition, we evaluated the prognostic value of SPINK1 expression across various cancer types. Our analysis revealed that high SPINK1 expression was associated with poor OS in glioblastoma, head and neck squamous cell carcinoma, clear cell renal cell carcinoma, papillary renal cell carcinoma, and mesothelioma (Fig. S7A). Differential expression of SPINK1 was observed in several cancer types, including esophageal carcinoma, head and neck squamous cell carcinoma, and hepatocellular carcinoma (Fig. S7B). Clinical-pathological characteristic analysis further indicated a strong correlation between SPINK1 expression and tumor grading in bladder urothelial carcinoma, head and neck squamous cell carcinoma, clear cell renal cell carcinoma, and papillary renal cell carcinoma (Fig. S7C). Considering the potential role of SPINK1 as a critical target for cancer immunotherapy, we performed a pan-cancer analysis of SPINK1 expression in relation to immune modulators. Our findings demonstrated a positive correlation between SPINK1 expression and various immune checkpoint inhibitors and immune stimulators across multiple tumor types (Fig. S7D). Lastly, drug sensitivity analysis revealed that patients with high SPINK1 expression exhibited increased sensitivity to vemurafenib and dabrafenib (Fig. S7E). Collectively, these results suggest that SPINK1 expression is closely associated with prognosis and tumor immunity in various cancers, highlighting its potential as a prognostic biomarker and therapeutic target in cancer immunotherapy.

### E4_SPINK1 interacts closely with CAFs to reshape the tumor microenvironment

**Fig. 5:**
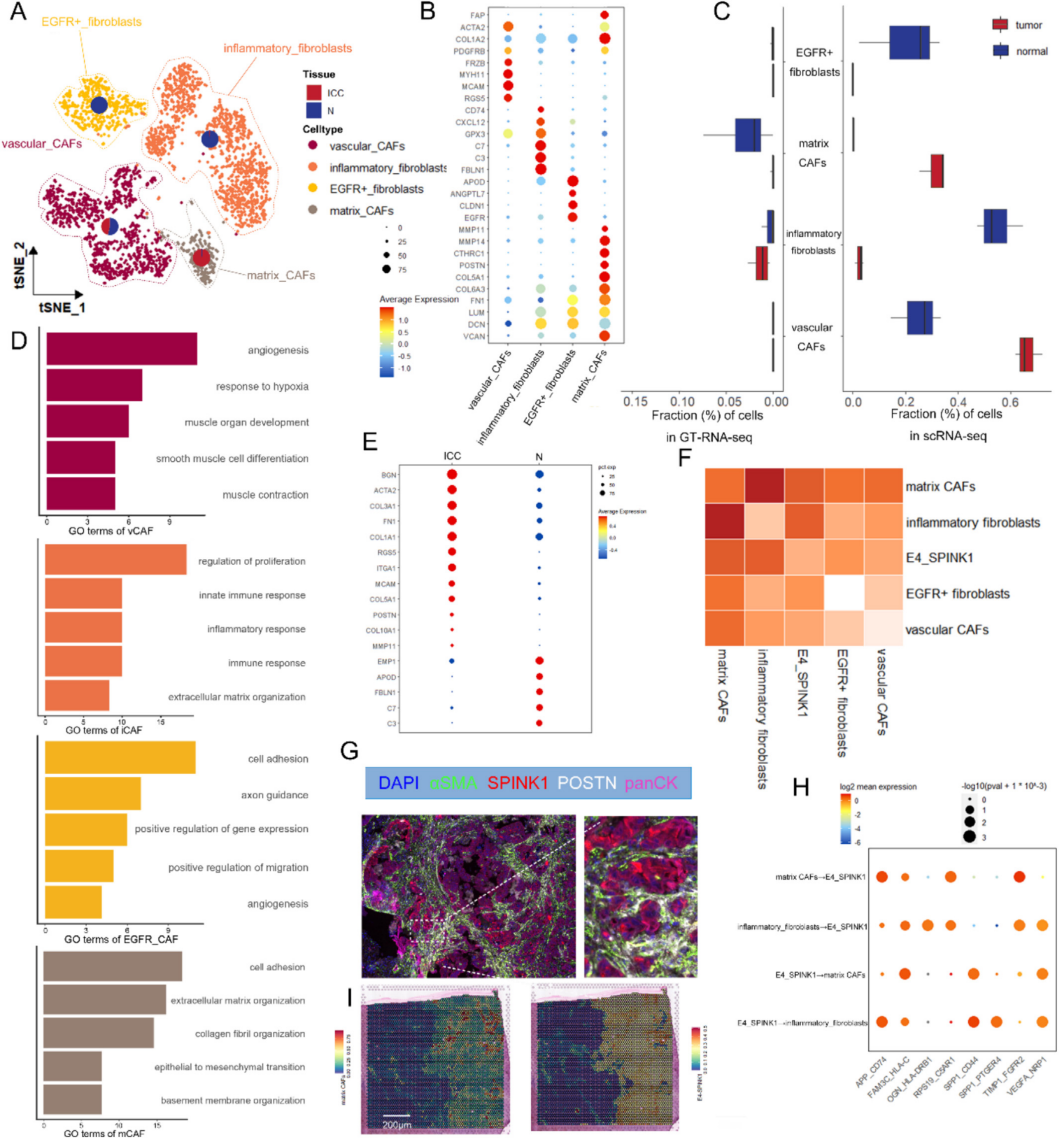
Transcriptional profiling of fibroblasts in the tumor microenvironment of ICC. A. tSNE plot showing the subtypes of cancer-associated fibroblasts (CAFs). B. Dot plot showing representative marker genes of CAFs. C. Bar plot showing the abundance of cell types in different CAFs subpopulations, based on scRNA-seq data from this study and deconvoluted bulk RNA-seq data from GEO & TCGA. D. Expression-based t-SNEs colored according to the AUC of each cell for the given gene-set. Shades of yellow/red are used when the cell AUC is greater than the assignment threshold, in shades of purple/black otherwise. E. Dot plot showing differentially expressed genes among CAFs subsets and in different tissues. F. Heatmap illustrating the cell–cell interaction patterns in E4_SPINK1 and CAFs. G. Immunofluorescent staining showing co-localization of αSMA (green), POSTN (white), SPINK1 (red), panCK (pink), and DAPI (blue) in ICC samples. H. Summary of selected ligand-receptor interactions from CellPhoneDB among E4_SPINK1, matrix_CAFs and inflammatory_fibroblasts. I. Co-localization of E4_SPINK1(top) and matrix_CAFs (bottom) revealed by spatial transcriptomics.

Fibroblasts are pivotal constituents of the TME and have attracted considerable research interest due to their roles in cancer progression. In the context of ICC, we identified four distinct fibroblast clusters, all exhibiting elevated expression of canonical fibroblast markers such as PDGFRB, COL1A2, DCN, and BGN (Supplementary Table 4; Fig. 5A). Based on differential gene expression profiles, we classified these clusters as cancer-associated fibroblasts (CAFs) and further subcategorized them. Matrix CAFs and vascular CAFs displayed high expression of ACTA2 and FAP, hallmark CAF markers (Fig. 5B). Vascular CAFs were distinguished by microvascular-specific genes, including ACTA2, MCAM, and MYH11, justifying their designation as vascular_CAFs. Inflammatory fibroblasts preferentially expressed immune-related genes such as FBLN1, C3, and C7, earning the label inflammatory_fibroblasts. Matrix CAFs were characterized by robust expression of extracellular matrix (ECM)-related genes, such as COL5A1, COL6A3, VCAN, and POSTN, supporting their classification as matrix_CAFs. Additionally, we identified a novel fibroblast subset with high EGFR expression, termed EGFR+_fibroblasts, which has not been previously documented in the literature.

We next assessed the infiltration patterns of these fibroblast clusters across tumor and adjacent tissues. Inflammatory_fibroblasts and EGFR+_fibroblasts were predominantly enriched in adjacent normal tissues, whereas matrix_CAFs and vascular_CAFs showed greater infiltration in ICC tumor tissues (Fig. 5C). Deconvolution analysis using the GT dataset corroborated these findings. To delineate their functional roles within the TME, we employed GO for gene set enrichment analysis. Inflammatory_fibroblasts were enriched in immune-related processes, including inflammatory response and immune response; matrix_CAFs exhibited enrichment in ECM and collagen fiber organization pathways, such as extracellular matrix organization and collagen fibril organization; and vascular_CAFs were associated with microvascular formation functions, including angiogenesis and muscle organ development (Fig. 5D). These results align with the expected biological roles of each subset.

Differential gene expression analysis across fibroblast clusters and tissue types revealed that FN1, ITGA1, COL3A1, and COL1A1 in matrix_CAFs, and RGS5, MCAM, and MMP11 in vascular_CAFs, were significantly upregulated in ICC tissues (Fig. 5E). This suggests that matrix_CAFs and vascular_CAFs may drive ICC occurrence and progression by remodeling the ECM and promoting microangiogenesis, respectively. To investigate potential interactions between E4_SPINK1—the most malignant ICC epithelial state—and fibroblasts, we utilized CellPhoneDB [40]. The analysis indicated a pronounced interaction between E4_SPINK1 and matrix_CAFs (Fig. 5F), which was validated by multiplex immunofluorescence (Fig. 5G). Ligand-receptor pairing identified FAM3C-HLA-C as the strongest interaction between E4_SPINK1 and matrix_CAFs (Fig. 5H). Prior studies have shown that FAM3C induces epithelial-mesenchymal transition (EMT) in cancer [46], a process critical for tumor invasion, metastasis, and recurrence. Given that functional enrichment analysis of ICC malignant cells highlighted ECM receptor interaction pathways (see Fig. 3E), we hypothesize that E4_SPINK1 promotes EMT via its interaction with matrix_CAFs, thereby enhancing tumor growth and invasiveness.

Furthermore, E4_SPINK1 exhibited high VEGFA expression, which binds to neuropilin-1 (NRP1) on matrix_CAFs, potentially driving angiogenesis and amplifying ICC’s proliferative and invasive capacity. Disrupting this VEGFA-NRP1 axis could hinder the complex vasculature formation essential for tumor expansion. Spatial transcriptomics further confirmed the physical proximity of E4_SPINK1 and matrix_CAFs within ICC tissues (Fig. 5I), providing a spatial foundation for their functional interactions.

### E4_SPINK1 recruits Lipid-Associated Macrophages to promote immunosuppression and ICC progression

**Fig. 6:**
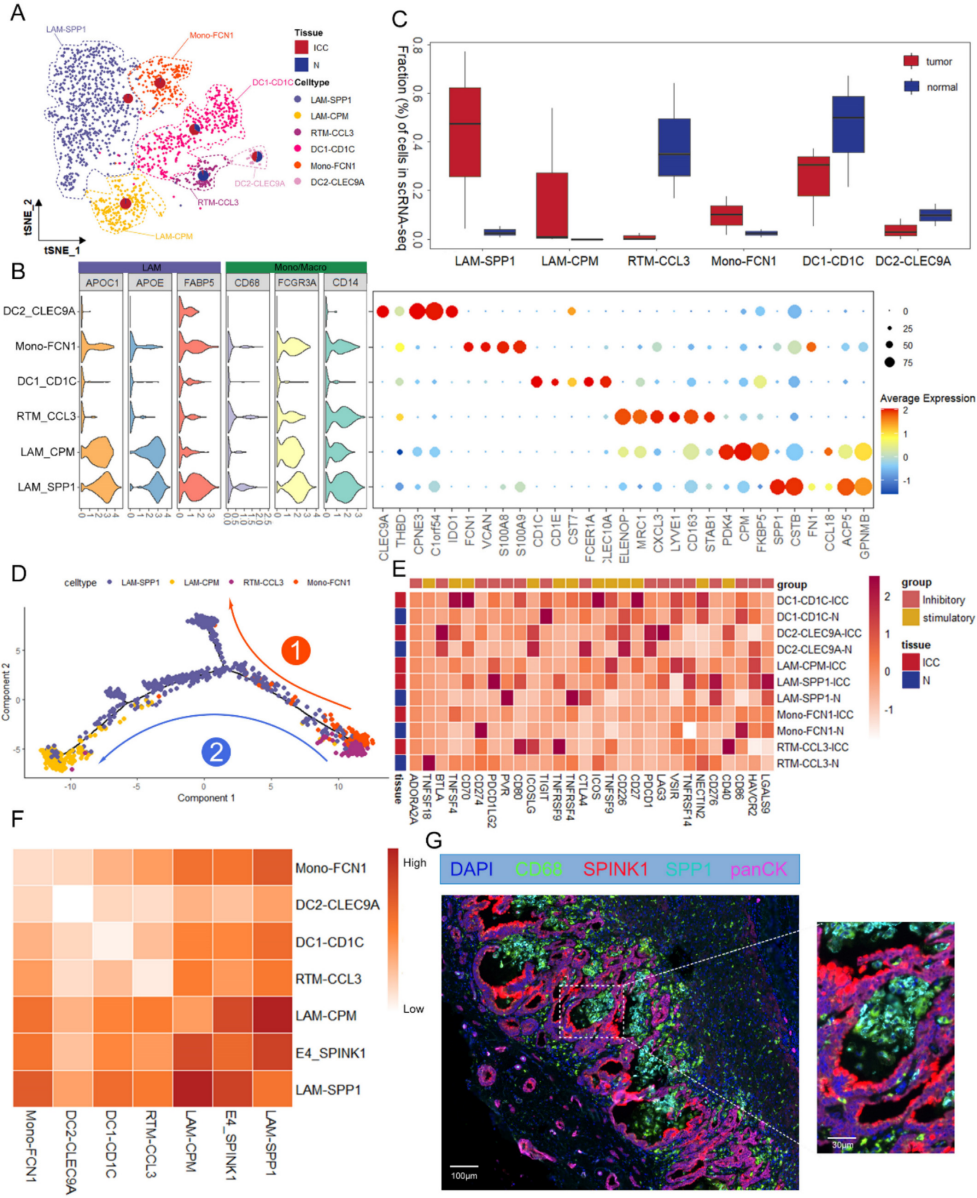
Transcriptional profiling of fibroblasts in the tumor microenvironment of ICC. A. tSNE plot showing the subtypes of Myeloid. B. Violin plots (left) displaying the representative expression pattern across different subtypes of myeloid. Dot plot (right) showing the expression of the top subtype-specific gene markers in each subtype. C. Bar plot showing the abundance of cell types in different Myeloid subpopulations, based on scRNA-seq data from this study. D. Semisupervised pseudotime trajectory of Monocytes and tumor-associated macrophages subtypes inferred by Monocle2. E. Heatmap showing the scaled expression levels of a series of immune checkpoint genes in myeloid cell subtypes. Subtypes are grouped by sample source and myeloid cell type annotations. Genes are grouped as receptor or ligand, inhibitory or stimulatory status and expected major lineage cell types known to express the gene (lymphocyte and myeloid). F. Heatmap illustrating the cell–cell interaction patterns in E4_SPINK1 and myeloid. G. Immunofluorescent staining showing co-localization of CD68 (green), SPP1 (cyan), SPINK1 (red), panCK (pink), and DAPI (blue) in ICC samples.

Given the established association between the E4_SPINK1 and ICC development, we sought to explore its interplay with the TME, focusing on myeloid cells due to their pronounced heterogeneity. Using specific marker genes (Supplementary Table 5), we re-clustered myeloid cells into six distinct subsets: one monocyte cluster, two dendritic cell (DC) clusters (DC1_CD1C and DC2_CLEC9A), and three macrophage clusters (RTM_CCL3, LAM_SPP1, and LAM_CPM) (Fig. 6A–B). Analysis of cell infiltration patterns revealed that DC1_CD1C and DC2_CLEC9A were significantly more abundant in adjacent normal tissues than in ICC tissues (Fig. 6C), suggesting their likely derivation from normal bile duct tissues. Similarly, RTM_CCL3 predominated in normal tissues, consistent with prior findings by Zhang et al. [47]. In contrast, the remaining clusters, notably the LAMs. LAM_SPP1 and LAM_CPM, exhibited greater infiltration in ICC tissues. These LAMs subsets, recently identified [48], highly expressed lipid metabolism genes such as APOE and APOC1 (Fig. S8A). To confirm their functional identity, we employed the AUCell package [49], which revealed enrichment in processes including lipid localization regulation, fatty acid transport, triglyceride metabolism, inflammatory responses, and immune system modulation (Fig. S8B), aligning with previous reports [50].

Focusing on LAMs due to their potential significance in the TME, we noted that LAM_SPP1 expressed high levels of SPP1 (osteopontin), a gene implicated in cancer progression and poor prognosis across multiple malignancies [51]. Similarly, LAM_CPM exhibited elevated CPM expression, previously linked to tumorigenesis [52]. Both LAM subsets also overexpressed CCL18, a chemokine associated with immunosuppression [53], with these genes showing heightened expression in ICC tissues within the GT dataset (Fig. S8C). These findings underscore the immunosuppressive and tumor-promoting roles of LAMs at the genetic level. Metabolic profiling using the scMetabolism package further revealed that LAMs in ICC tissues predominantly utilized lipid metabolism, contrasting with the glucose- and amino acid-dominated metabolism in normal tissues (Fig. S8D). Developmental trajectory analysis with Monocle2 [54] positioned LAM_SPP1 and LAM_CPM as endpoints of two primary differentiation branches within the monocyte-macrophage lineage, reinforcing their critical involvement in ICC progression (Fig. 6D).

Recognizing myeloid cells as key sources of immune checkpoints [55], we examined their checkpoint expression profiles. Myeloid cells infiltrating tumor tissues, particularly LAM_SPP1, upregulated inhibitory checkpoints such as PDCD1LG2, PDCD1, LAG3, VSIR, TNFRSF14, CD276, HAVCR2, and LGALS9 (Fig. 6E). Conversely, myeloid cells in adjacent tissues expressed co-stimulatory checkpoints, including TNFSF18, ICOSLG, and TNFRSF4. These data suggest that LAM_SPP1 may foster tumorigenesis by promoting an immunosuppressive TME.

To investigate interactions between E4_SPINK1—the most malignant ICC tumor cell state—and myeloid cells, we utilized CellPhoneDB to identified the strongest interactions between E4_SPINK1 and LAMs [56], particularly LAM_SPP1 (Fig. 5F–G). Ligand-receptor pairing analysis pinpointed APP-CD74 as the most robust interaction between E4_SPINK1 and LAM_SPP1. We propose that ICC recruits LAM_SPP1 to the tumor via amyloid precursor protein (APP) on E4_SPINK1 binding to the CD74 receptor on LAM_SPP1, potentially inhibiting macrophage phagocytosis through the APP-CD74 axis, akin to mechanisms observed in glioblastoma [57] (Fig. S8E). Additionally, we identified RPS19-C5aR1 as another key interaction, where E4_SPINK1-derived ribosomal protein S19 (RPS19) engages complement C5a receptor 1 (C5aR1) on LAM_SPP1, mirroring tumor-promoting mechanisms in breast and ovarian cancers [58]. The SPP1-CD44 pair, highly expressed in both E4_SPINK1 and LAM_SPP1, has been reported to suppress T-cell proliferation in ICC [59]. We also observed the SPP1-CD44 interaction between E4_SPINK1 and matrix_CAFs (see Fig. 5H), suggesting a conserved mechanism of tumor promotion across TME cell types. Emerging evidence suggests that pathologically significant crosstalk between LAMs and the E4_SPINK1 molecular axis may orchestrate the tumorigenesis and progression of ICC through bidirectional molecular interplay.

### E4_SPINK1 recruits LAMs infiltration via CCL20 secretion

**Fig. 7:**
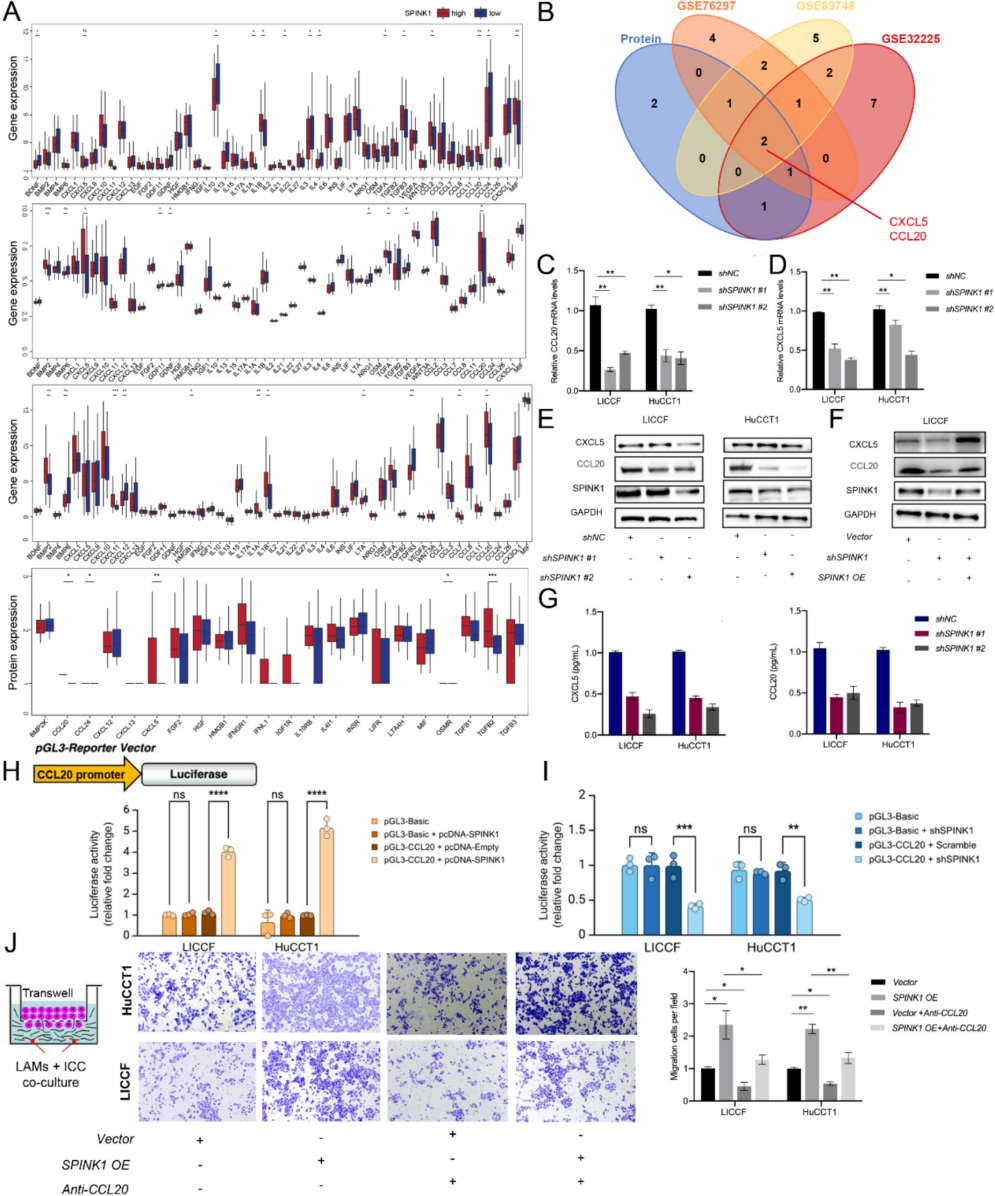
E4_SPINK1 Recruits LAMs Infiltration via CCL20 Secretion A. The differential expression of SPINK1 and common cytokines and chemokines across four cohorts was analyzed. The cohorts, listed from top to bottom, include GSE32225, GSE76297, GSE89748, and our own proteomic validation cohort. B. Cross-cohort analysis of the four study groups. C-D. Quantitative PCR (qPCR) analysis of CCL20 (C) and CXCL5 (D) expression in SPINK1-silenced ICC cells. E. Western blotting analysis revealed that silencing SPINK1 leads to a downregulation of CCL20 and CXCL5 expression in ICC cells. F. Western blotting analysis indicated that overexpression of SPINK1 significantly increases the expression levels of CCL20 and CXCL5 in ICC cells. G. ELISA results demonstrated that silencing SPINK1 significantly reduces the protein levels of CCL20 and CXCL5 in ICC cells. H. Luciferase reporter assays demonstrated that SPINK1 is capable of binding to the CCL20 promoter. I. Luciferase reporter assays demonstrated that silencing SPINK1 significantly diminished its binding to the CCL20 promoter. J. Transwell assays showed that SPINK1 overexpression enhances the migratory and invasive capacities of ICC cells within a co-culture system with LAMs. Additionally, the introduction of CCL20 inhibited the migration and invasion of tumor cells.

To further elucidate the molecular mechanisms underlying this crosstalk, we turned to chemokines—secreted signaling molecules known to regulate cell recruitment, immune responses, and tumor progression [60,61]. Among these, CCL20 (C–C motif chemokine ligand 20) emerged as a compelling candidate due to its documented roles in cancer [62]. Based on SPINK1 expression levels, we stratified GEO datasets (GSE32225, GSE76297, GSE89748) and our proteomic cohort into high and low SPINK1 expression groups to explore the relationship between SPINK1 and common cytokines or chemokines (Fig. 7A). Across all four datasets, we consistently found that high SPINK1 expression was significantly associated with elevated levels of CCL20 and CXCL5 (Fig. 7B). After validation by qPCR, we observed that silencing SPINK1 resulted in decreased expression of CCL20 and CXCL5 (Fig. 7C–D). Subsequently, we confirmed this observation using Western blot analysis, which revealed a reduction in the protein levels of CCL20 and CXCL5 in ICC cells following SPINK1 silencing. This effect was reversed upon overexpression of SPINK1 (Fig. 7E–F).

Additionally, we collected the supernatant from SPINK1-silenced ICC cells for ELISA. The results demonstrated that silencing SPINK1 led to a reduction in the protein levels of CCL20 and CXCL5 (Fig. 7G). Subsequent luciferase reporter assay results demonstrated that SPINK1 enhanced the luciferase activity of the CCL20 promoter, indicating that SPINK1 may bind to the CCL20 promoter to facilitate CCL20 transcription, the phenomenon was suppressed upon SPINK1 silencing (Fig. 7H–I). Previous studies have reported that glioblastoma cells can promote macrophage infiltration via CCL20 secretion [63]. This led us to hypothesize that E4_SPINK1 may promote CCL20 secretion via SPINK1, thereby facilitating the recruitment of LAMs. To investigate the role of CCL20 in E4_SPINK1-mediated LAMs infiltration, we co-cultured ICC cells with LAMs and performed Transwell assays. The results demonstrated that the migration and invasion abilities of ICC cells were significantly reduced following the addition of CCL20 neutralizing antibody. Upon administration of CCL20-neutralizing antibody to SPINK1-overexpressing ICC cells, a similar phenomenon was observed (Fig. 7J). In summary, these findings strongly suggest that SPINK1 promotes ICC progression by recruiting LAMs via CCL20 chemotaxis.

We reveal that CCL20 plays a critical mediator role within the SPINK1-TME network, linking epithelial malignancies with stromal and immune modulation to drive ICC progression. Our findings reveal how SPINK1 exploits the dynamic changes in the TME—through LAMs, CAFs, and now CCL20—to sustain the aggressive phenotype of ICC, providing new avenues for research and intervention.

### Lams facilitate glutamine Provision to E4_SPINK1 to support glutamine metabolism

**Fig. 8:**
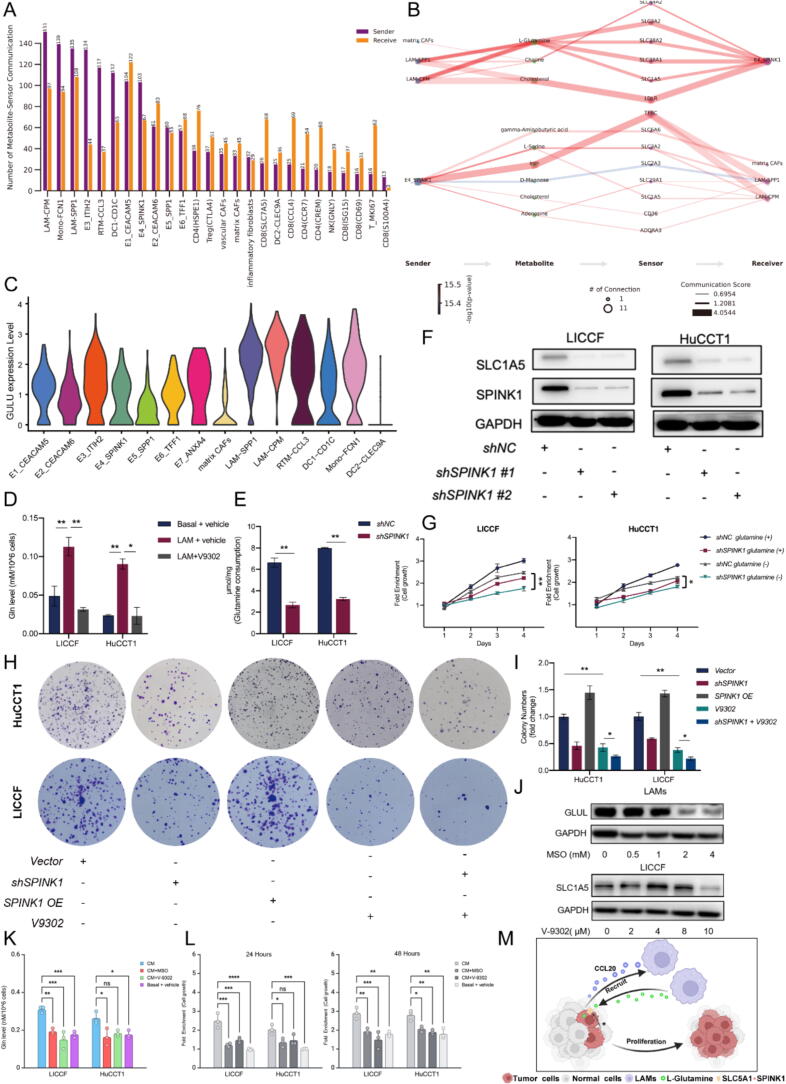
LAMs Supply Glutamine to E4_SPINK1 for Glutamine Metabolism A. Bar plot illustrating the communication frequency between sender and receiver. The x-axis represents the cell subtypes, while the y-axis indicates the number of communications. Orange and purple bars represent the communication frequencies of the sender and the receiver, respectively. B. Illustration of metabolite-sensor communication through the information flow between metabolites and sensors. The size of the nodes reflects the number of connections. These lines connect the sender, metabolites, sensors, and receiver. The color of the lines represents the − log10(p-value), and the width of the lines indicates the communication score. C. Violin plot showing the mRNA expression levels of GLUL across different cell subtypes. D. Intracellular Gln levels in ICC cells following treatment with V9302 and co-culture with LAMs. E. Glutamine consumption capacity of ICC cells following SPINK1 silencing. F. The effect of SPINK1 depletion (shSPINK1) on the expression levels of SLC1A5. G. The impact of SPINK1 depletion (shSPINK1) and glutamine on the growth capacity of ICC cells. H-I. Colony formation assays in ICC cells indicate that overexpression of SPINK1 promotes ICC proliferation, while V9302 treatment inhibits ICC proliferation. J. The expression levels of GLUL in LAMs and SLC1A5 in LICCF were significantly downregulated following treatment with MSO and V9302. K. Intracellular gln levels of ICC cells after MSO, V9302 and conditional medium treatment. L. Cell growth of ICC cells treated with MSO, V9302 and conditional medium after 24 h (Left) or 48 h (Right). M. E4_SPINK1 interacting with LAMs to promote metabolic reprogramming in TME.

To systematically interrogate the metabolic crosstalk within the tumor microenvironment (TME) of intrahepatic cholangiocarcinoma (ICC), we employed the MEBOCOST computational framework to analyze metabolite sensor-mediated interactions among E4_SPINK1 [64], LAMs and matrix_CAFs. Our analysis elucidated a pronounced communication network between E4_SPINK1 and LAMs (Fig. 8A), with quantitative assessment revealing that the interaction scores for L-glutamine and its associated transporters significantly surpassed those of other metabolites (Fig. 8B). This finding suggests a preferential metabolic dependency on glutamine in the E4_SPINK1-LAMs axis. Corroborating this, scRNA-seq data demonstrated a markedly elevated expression of GLUL—the gene encoding glutamine synthetase, which catalyzes the conversion of glutamate to glutamine—in LAMs compared to other cellular constituents of the TME (Fig. 8C). In contrast, GLUL expression in E4_SPINK1 was notably diminished, indicating a limited endogenous capacity for glutamine biosynthesis and underscoring a potential reliance on exogenous glutamine supply from LAMs.

To substantiate these computational insights, we conducted experimental validation by quantifying intracellular glutamine levels in ICC cells under controlled co-culture conditions with LAMs. The results revealed a significant augmentation of glutamine content in ICC cells when co-cultured with LAMs (Fig. 8D), an effect abrogated by treatment with V-9302, a selective inhibitor of the glutamine transporter ASCT2 (SLC1A5). This inhibition precipitated a substantial reduction in intracellular glutamine levels, confirming the role of SLC1A5 in mediating glutamine uptake. Furthermore, silencing SPINK1 in ICC cells resulted in a pronounced downregulation of glutamine consumption (Fig. 8E), suggesting that SPINK1 modulates the metabolic interplay between ICC cells and LAMs by regulating glutamine transport dynamics. Western blot analysis further corroborated this hypothesis, revealing a concomitant decrease in SLC1A5 protein expression following SPINK1 knockdown (Fig. 8F), thereby linking SPINK1 expression to the molecular machinery of glutamine uptake.

To explore the functional implications of this metabolic dependency, we supplemented the culture medium of ICC cells with exogenous glutamine, observing a significant enhancement of tumor cell proliferation (Fig. 8G). This growth-promoting effect was notably attenuated upon SPINK1 silencing, underscoring the pivotal role of SPINK1 in glutamine-driven oncogenesis. Moreover, the addition of V-9302 in the context of SPINK1 silencing synergistically amplified the inhibition of tumor cell proliferation (Fig. 8H–I), providing compelling evidence that SPINK1 orchestrates ICC progression by facilitating glutamine influx via SLC1A5. Furthermore, LAMs were cultured in complete medium, and the supernatant was collected as conditioned medium after the removal of LAMs. ICC cells were then cultured in the conditioned medium with the addition of MSO (L-methionine sulfoximine, a glutamine synthetase inhibitor) or V9320. We observed a pronounced downregulation of GLUL expression in LAMs and SLC1A5 expression in LICCF following treatment with MSO and V9302 (Fig. 8J). Notably, compared to ICC cells cultured in control medium, those cultured in conditioned medium exhibited significantly elevated intracellular glutamine levels and enhanced cell proliferation (Fig. 8K–L). However, both effects were markedly suppressed upon the addition of MSO or V9320. Collectively, these data delineate a metabolic paradigm wherein LAMs serve as an exogenous source of glutamine synthesis, mediated by elevated GLUL activity, and transfer this critical metabolite to E4_SPINK1 cells. In turn, E4_SPINK1 upregulates SPINK1 expression to enhance SLC1A5-dependent glutamine uptake, thereby fueling glutamine metabolism and sustaining ICC malignancy (Fig. 8M). This bidirectional metabolic symbiosis highlights a novel therapeutic vulnerability in ICC, potentially exploitable through targeted inhibition of glutamine transport or synthesis pathways.

## Discussion

ICC poses significant therapeutic challenges, primarily due to its pronounced intratumor heterogeneity and a complex TME enriched with desmoplastic stroma [65]. Despite this, only a few studies have investigated the cellular ecosystem in ICC and their interactions with TME components using single-cell sequencing approaches [66,67]. By integrating scRNA-seq with multi-omics analysis, this study systematically elucidates the molecular mechanism whereby E4_SPINK1 tumor cells recruit LAMs via CCL20 secretion and rely on LAMs-derived glutamine to meet their metabolic demands. This finding not only enhances our understanding of metabolic reprogramming in ICC but also offers novel insights into potential therapeutic strategies.

Our results establish E4_SPINK1 as a highly malignant tumor cell cluster in ICC, with its elevated infiltration strongly correlated with poor patient prognosis. Pseudotime trajectory analysis further confirms that E4_SPINK1 represents the endpoint of tumor cell evolution, underscoring its pivotal role in ICC progression. Notably, glutamine metabolism is critical for tumor cell bioenergetics and biosynthesis [68,69]. We demonstrate that LAMs synthesize glutamine via elevated GLUL expression and transfer it to E4_SPINK1 [70], and E4_SPINK1 enhances glutamine uptake by upregulating SPINK1 and SLC1A5. This bidirectional metabolic symbiosis fuels the rapid proliferation and invasiveness of ICC [71]. Experimental evidence supports this, showing that inhibition of SLC1A5 or silencing of SPINK1 markedly impairs ICC cell proliferation, suggesting the glutamine metabolism axis as a promising therapeutic target. Additionally, glutamine may further promote ICC progression by suppressing ferroptosis, a mechanism warranting further investigation [72].

Within the immune microenvironment, LAMs in ICC tissues exhibit pronounced lipid catabolism, contrasting with lipid biosynthesis observed in adjacent normal tissues. We hypothesize that E4_SPINK1 may modulate LAMs’ lipid catabolism, utilizing the resulting metabolites to support its own lipid synthesis, thereby reinforcing the immunosuppressive function of LAMs. This lipid metabolism synergy likely diminishes tumor immunogenicity, aligning with prior reports linking lipid catabolism to immune tolerance [73]. Concurrently, CellphoneDB analysis reveals robust interactions between E4_SPINK1 and LAM-SPP1, with CD74 emerging as a frequently enriched receptor. Elevated CD74 expression may inhibit antigen presentation by blocking peptide binding to T cells, suggesting its potential as a target for ICC immunotherapy [74].

Moreover, the interplay between E4_SPINK1 and matrix cancer-associated fibroblasts (matrix_CAFs) merits attention. Matrix_CAFs, enriched in extracellular matrix (ECM) remodeling and collagen-related pathways, are key contributors to the fibrotic stroma associated with adverse clinical outcomes in ICC [75]. Our findings identify FAM3C-HLA-C and SPP1-CD44 as critical ligand-receptor pairs mediating E4_SPINK1-matrix_CAFs interactions. We propose that FAM3C may induce EMT in E4_SPINK1, exacerbating the desmoplastic reaction, while the widespread enrichment of the SPP1-CD44 axis suggests it as a central node in TME communication. Disrupting SPP1-CD44 could simultaneously impair interactions with both LAMs and matrix_CAFs, offering a potential comprehensive therapeutic approach for ICC.

The recruitment of LAMs by E4_SPINK1 via CCL20 further highlights its role in shaping an immunosuppressive TME. Consistent with reports of CCL20 recruiting M2 macrophages in breast cancer [76], we observed a significant positive correlation between SPINK1 expression and CCL20 levels, indicating that E4_SPINK1 employs this pathway to modulate LAMs’ metabolic reprogramming. This dual regulation of metabolism and immunity likely drives ICC initiation and progression. Leveraging differentially expressed genes from E4_SPINK1, including ATP1A1, CP, and SPINK1, we developed a GBM-based risk prediction model with robust prognostic accuracy, providing a novel tool for personalized ICC management. However, the specific roles of genes like ATP1A1 and CP in ICC remain to be elucidated in future studies.

Our study has certain limitations. The foremost limitation is the relatively small sample size used for single-cell sequencing. Although we have constructed independent validation cohorts by integrating samples from multiple public databases, including TCGA and GEO, and have employed multicenter proteomic validation cohorts as well as multi-omics analyses to ensure the robustness of our results, we acknowledge that larger-scale, prospective, multicenter cohort studies are necessary to ultimately translate SPINK1 into clinical application. In the future, we aim to pursue such collaborative efforts to further consolidate the conclusions of this study. Additionally, another limitation of this study is that, although we identified molecules such as ATP1A1 and CP with significant prognostic value through bioinformatics analyses, their specific functional mechanisms in ICC were not elucidated in the current work. In future studies, we plan to investigate the precise roles of these and other factors significantly associated with poor prognosis in ICC beyond SPINK1, thereby further deepening our understanding of the ICC. In addition, this study identified potential therapeutic vulnerabilities, such as SPP1-CD44 and CCL20-CCR6, through bioinformatics analysis. Although these predictions have been supported by spatial transcriptomics and some functional experiments, their therapeutic efficacy in vivo still needs to be validated through antibody blocking, small molecule inhibitors, and other approaches in preclinical models. This will be a key focus of our future research, aimed at translating the mechanistic discoveries of this study into viable therapeutic strategies.

In conclusion, our study provides an in-depth description of the transcriptome and functional phenotypes of multiple cellular components of the tumor microenvironment in the development and progression of ICC. And we built a model to predict patient prognosis based on its differential expressed genes. In addition, we also found that there were close cellular interactions between E4_SPINK1 and various components in the tumor microenvironment. We further identified that E4_SPINK1 can recruit LAMs via CCL20 to induce metabolic reprogramming.

## Conclusion

This study comprehensively delineates the transcriptomic profiles and functional phenotypes of multiple TME components in ICC, unveiling a mechanism whereby E4_SPINK1 recruits LAMs via CCL20 and relies on their glutamine supply for metabolic reprogramming. The risk prediction model based on E4_SPINK1 differentially expressed genes offers a reliable tool for prognostic assessment. The intricate interactions between E4_SPINK1, LAMs, and matrix_CAFs suggest that targeting glutamine metabolism or key ligand-receptor pairs could represent a promising avenue for precision therapy in ICC.

## Data availability

The public datasets used in this study, including the dataset of Ahn KS et al. (including 30 tumor samples and 27 normal samples), were retrieved from GSE107943. The TCGA PAAD dataset was obtained from the GDC data portal (https://portal.gdc.cancer.gov/projects/TCGA-PAAD). Spatial transcriptomics data were obtained from the Genome Sequence Archive under the accession number HRA000437(77).

## Compliance with Ethics Requirements

This work did not involve any human participants. All animal experiments were reviewed and provisionally approved by the Clinical Research Ethics Committee of the First Affiliated Hospital, Zhejiang University School of Medicine. All procedures involving animals were conducted in strict accordance with the Guidelines for the Care and Use of Laboratory Animals of the National Institutes of Health and the ARRIVE guidelines for reporting animal research, ensuring humane treatment and minimizing suffering throughout the study.

## Studies with human subjects

All procedures followed were in accordance with the ethical standards of the responsible committee on human experimentation (institutional and national) and with the Helsinki Declaration of 1975, as revised in 2008 (5). Informed consent was obtained from all patients for being included in the study.

The collection and use of all surgical specimens were approved by the Ethics Committee of the Affiliated Hospital of Southwest Medical University, with approval number ky2025264. In addition, all patients provided written informed consent prior to participation.

## Credit Author Statement

X.R. and J.R. conceived the study, designed experiments, and supervised the project. J.G. and C.Y performed key functional experiments, including single-cell RNA sequencing (scRNA-seq) analysis, in vitro assays and animal model validation. Q.L. and M.W. contributed to data collection, bioinformatics analysis, and metabolic profiling. W.F. and J.C. conducted proteomic analysis, immunohistochemistry, and multiplex immunofluorescence experiments. D.Z. and R.C. curated clinical samples, performed survival analysis, and correlated molecular findings with patient outcomes. G.S, R.Z. and Y.J. drafted the manuscript, integrated revisions, and coordinated interdisciplinary collaboration. All authors critically reviewed, edited, and approved the final version of the manuscript

## Funding

This work was supported by the “Pioneer” and “Leading Goose” R&D Program of Zhejiang (2024C03175), Beijing Science and Technology Innovation Medical Development Foundation (KC2023-JX-0186-FZ099), National Natural Science Foundation of China (82473004, 81874173, 82073375, 82573368), Zhejiang Provincial Natural Science Foundation of China (LY22H160019, MS25H160091), Beijing Xisike Clinical Oncology Research Foundation (Y-MSDZD2022-0161), GuangDong Basic and Applied Basic Research Foundation (2025A1515012516 to X. Rong; 2025A1515012253 to R. Zhou).

## Declaration of competing interest

The authors declare that they have no known competing financial interests or personal relationships that could have appeared to influence the work reported in this paper.
