# Supplementary material for: SPINK1-driven cellular heterogeneity and interaction networks in intrahepatic cholangiocarcinoma revealed by integrated multi-omics analysis
Source: J Adv Res. 2025 Nov 17;86:573–90. doi: 10.1016/j.jare.2025.11.011 (PMC13453628; doi:10.1016/j.jare.2025.11.011)
Supplement: Supplementary file 1 [file mmc1.docx]

**Figure S1. Cluster tree of cell subpopulation.** Cluster tree of cell subpopulation (**A.** All cell types, **B.** Epithelial, **C.** Fibroblasts, **D.** Myeloid).

**Figure S2. representative marker genes across cell clusters.**

**A.** Harmony method was employed to correct for batch effects. **B.** tSNE plots showing the expression of selected canonical marker genes for all cell clusters.

**Figure S3. Copy Number Variation analysis of** **epithelial cells.**

**A.** Heatmap showing the predicted CNV level in the epithelial cells. The normalized CNV levels were shown, the red color represents high CNV level and blue represents low CNV level. **B.** Joyplots show the distribution of CNV score in different samples. **C.** Bar plot showing the abundance of cell types in different epithelial cells subpopulations, based on scRNA-seq data from this study and deconvoluted bulk RNA-seq data from GEO & TCGA

**Figure S4. Survival analysis of different epithelial cells subsets.**

**A-B.** Kaplan–Meier curves of GEO&TCGA ICC patients (n = 58) showing the Overall survival(A) and Disease-free survival(B) grouped by the cell abundance in different epithelial cells subsets.

**Figure S5. Malignancy of epithelial cells.**

**A.** Expression of different epithelial cells subsets in GEO&TCGA ICC samples (n = 58) with different clinical stages. **B.** Gene set enrichment analysis in each cell cluster.

**Figure S6. Validations of findings of E4_SPINK1 in large cohort samples.**

**A.** Dot plot showing the metabolic activity analysis of all epithelial cells subclusters in different tissue by scMetabolism. **B.** Dot plots showing the expression of highly expressed E4_SPINK1 genes in the epithelial cells subsets, scRNA-seq dataset using GSE125449. **C.** Kaplan–Meier curves of Proteomics cohort (n=75) showing the Overall survival and Disease-free survival grouped by the expression of SPINK1. **D.** Expression of SPINK in Proteomics cohort ICC samples (n = 67) with different clinical stages. E**.** Building predictive models based on machine learning methods. **F.** The effectiveness of the prediction model tested in TCGA (top) and GEO (bottom) datasets.

**Figure S7:** **SPINK1 pan-carcinoma analysis.**

**A.** Prognostic analysis of SPINK1 and tumor. **B.** The expression level and clinical staging of SPINK1 in tumor and normal tissues. **C.** Clinical staging of SPINK1 in tumor and normal tissues. **D.** Analysis of differences between SPINK1 expression and tumor immunostimulatory, immunoinhibitor. **E.** Analysis of SPINK1 expression and tumor drug sensitivity. The numbers within the red circles represent the adjusted p-values. All p-values have been adjusted for multiple hypothesis testing using the Bonferroni correction. Statistical significance thresholds are as follows: * *p* < 0.05; ** *p* < 0.01; *** *p* < 0.001.

**Figure S8. The transcriptional landscape of myeloid cells in ICC microenvironment.**

**A.** tSNE plots showing the expression of selected canonical marker genes for all cell clusters. **B.** The bar plot shows the AUC score for each cell cluster. **C.** The expression level of CCL18, CPM, SPP1 in the GEO&TCGA dataset. **D.** Dot plot showing the metabolic activity analysis of all myeloid subclusters in different samples by scMetabolism. **E.** Summary of selected ligand-receptor interactions from CellPhoneDB among E4_SPINK1, LAM-CPM and LAM-SPP1.
