# Supplementary material for: SPINK1-driven cellular heterogeneity and interaction networks in intrahepatic cholangiocarcinoma revealed by integrated multi-omics analysis
Source: J Adv Res. 2025 Nov 17;86:573–90. doi: 10.1016/j.jare.2025.11.011 (PMC13453628; doi:10.1016/j.jare.2025.11.011)
Supplement: Supplementary file 2 [file mmc2.docx]

**Tissue processing and cell Isolation**

Freshly excised ICC and adjacent tissues were immediately placed in RPMI-1640 medium (Gibco; cat. no. 11875-093) supplemented with 1 mmol/L protease inhibitor (Solarbio; cat. no. P6730) and transported to the research facility. Tissue samples were digested using 2 mg/mL Dispase II (Sigma-Aldrich; cat. no. 42613-33-2), 1 mg/mL Type VIII collagenase (Sigma-Aldrich; cat. no. C2139), and 1 unit/mL DNase I (NEB, cat. no. M0303S) in phosphate-buffered saline (PBS) with 5% fetal bovine serum (FBS; Gibco; cat. no. 16000-044) at 37°C for 40 minutes. Cells were mechanically dissociated by vortexing and pipetting at 20-minute intervals, then filtered through a 40-µm nylon strainer (Falcon; cat. no. 352340). Erythrocytes were lysed using red blood cell lysis buffer (Invitrogen) containing 1 unit/mL DNase I. The resultant cell suspensions were washed with PBS containing 0.04% bovine serum albumin (BSA; Sigma-Aldrich; cat. no. B2064). Cell concentration was determined using the Countes (Thermo) platform and adjusted to 1,000 cells/mL. Single cells were loaded onto the Chromium platform (V2 chemistry) according to the manufacturer’s protocol to capture 5,000–10,000 cells per chip position. Subsequent library preparation followed the standard Chromium protocol.

**Trajectory analysis**

We used Monocle2 (version 2.18.0) to infer the cell lineage trajectory. The “setOrderingFilter” function was applied to sort the cells (cutoff value was q < 0.001) for the variable genes identified by the “differentialGeneTest” function. Finally, dimensionality reduction was performed using the “reduceDimension” function (using the “DDRTree” dimensionality reduction method). The visualization function “plot_cell_trajectory” was used to plot the pseudo-time trajectories of each cell lineage.

**Cell–cell interaction analysis**

Cell–cell interactions among the cell types were estimated by CellPhoneDB (v4.0.0) and using the version 2.0.0 of the database. CellPhoneDB infers the potential interaction strength between two cell subsets based on the gene expression level of a receptor-receptor pair. The significance of interaction is assessed through a permutation test (1000 times). Enriched ligand-receptor interactions between two cell subsets were calculated based on permutation tests. We extracted significant ligand-receptor pairs with P values <0.01.

**Protein extraction and Western blot analysis**

Initially, total protein was extracted from the LICCF and HuCCT1 cell lines and subsequently quantified to ensure accurate protein loading. The protein samples were then subjected to electrophoresis on an SDS-PAGE gel for effective separation based on their molecular weights. Following electrophoresis, the separated proteins were transferred onto a PVDF membrane for improved antibody accessibility and detection sensitivity. To minimize non-specific antibody binding, the membrane was blocked with an appropriate blocking solution. Next, the membrane was incubated with specific primary antibodies targeting SPINK1(1:1000, Cellsignal,12071S) and GAPDH (1:500, Santa Cruz, sc-47724) (as a loading control) to recognize and bind the target proteins. Following primary antibody incubation, a horseradish peroxidase (HRP)-conjugated secondary antibody was applied to amplify the signal from the bound primary antibodies. Finally, chemiluminescence was utilized to detect the expression levels of the target proteins, and the resulting signals were captured and analyzed using an advanced imaging system to ensure accurate and reproducible quantification. The signals were detected with an ECL Kit (Bio-Rad, ClarityTM Western ECL Substrate, 500 ml #1705061).

**CCK8 Assay**

LICCF and HuCCT1 cell lines were inoculated into a 96-well plate at a density of 5000 cells per well, and then treated accordingly based on the experimental design (transfected with control plasmids or plasmids shRNA for SPINK1 knockdown) (SPINK1 Human shRNA Plasmid Kit, Origene, 6690). At specified time points (days 0, 1, 2, 3, and 4), CCK8 reagent (Abcam, ab228554)) was added to each well, and the plate was further incubated for a certain period. Subsequently, the absorbance at 450 nm was measured using a microplate reader.

**Colony Formation Assay**

LICCF and HuCCT1 cell lines treated accordingly based on the experimental design (transfected with control plasmids or plasmids shRNA for SPINK1 knockdown) (500 cells per dish) were inoculated into appropriate culture dishes and cultivated under standard conditions (37°C, 5% CO2) for a specified period. During this time, the medium was regularly replaced with fresh one based on cell growth observations. Cell proliferation was monitored for 10 to 14 days, and the culture was terminated once clearly visible colonies had formed. Subsequently, the cells were fixed with formaldehyde to preserve the colony morphology, followed by staining with crystal violet solution (Sigma-Aldrich, V5265) to enhance the visibility of the colonies. After thorough washing to remove any excess dye, the culture dishes were allowed to dry. The number of colonies was counted under a microscope, and statistical analysis was performed.

**Transwell and Migration Assay**

A predetermined number of cholangiocarcinoma cells (LICCF and HuCCT1) treated accordingly based on the experimental design (transfected with control plasmids or plasmids shRNA for SPINK1 knockdown, or co-cultured with LAMs) (500,000 cells per Transwell insert), were seeded into the upper chamber of a Transwell® system (Sigma-Aldrich, CLS3414-24EA), consisting of an insert with an 8.0 µm pore size polycarbonate membrane separating the upper and lower chambers. For the migration assay, the upper chamber was filled with 200 µL of serum-free culture medium, ensuring that no growth factors or other chemoattractants were present. In contrast, the lower chamber contained 600 µL of complete culture medium supplemented with 10% FBS, acting as a chemoattractant to stimulate cell migration. After culturing for a specific duration (48 hours), non-migratory or non-invasive cells on the upper surface of the membrane were removed using a cotton swab. The cells that had successfully migrated or invaded through the membrane pores and adhered to the underside of the membrane were then fixed with 4% paraformaldehyde for 15-30 minutes, followed by staining with 0.1% crystal violet solution for 30 minutes to enhance their visibility. After staining, the membranes were thoroughly rinsed with distilled water to remove excess crystal violet. The Transwell inserts were then cut out and mounted onto glass slides with a drop of mounting medium. The number of migrated or invaded cells was counted manually using a light microscope at a magnification of 100 x or 200 x in multiple fields (at least five fields per membrane).

**Flow Cytometry for Cell Apoptosis Analysis**

The LICCF and HuCCT1 cells transfected with the control and SPINK1 knockout plasmids were collected and washed to eliminate impurities from the culture medium. Following the Annexin V-FITC/PI Apoptosis Detection Kit instructions (BD 556547 AnnexinV-FITC/PI Assay Kit), the washed cells were resuspended in an appropriate volume of staining solution, gently mixed with a pipette to ensure all cells were exposed to the dye, and incubated under light-blocking conditions for 15-20 minutes to allow complete dye binding. Detection was performed using a flow cytometer, with appropriate voltage and compensation parameters set to ensure clear distinction between the fluorescent signals of Annexin V-FITC and PI. Sufficient data points were collected for statistical analysis, and the detection data were analyzed using the flow cytometer's built-in software or third-party data analysis software. Based on the fluorescent signals, the cells were categorized into four subpopulations: live cells (Annexin V-/PI-), early apoptotic cells (Annexin V+/PI-), late apoptotic cells (Annexin V+/PI+), and necrotic cells (Annexin V-/PI+). The proportions of apoptotic cells (including both early and late apoptotic cells) were calculated and compared between treatment groups to evaluate the treatment effect.

**Immunohistochemical staining**

We selected appropriate ICC tissue samples, ensuring they were fresh and had not undergone freezing or other treatments to maintain antigen activity. Slides were stained with SPINK1 antibodies using the GTvisionIII Immunohistochemical Assay Kit (HRP/DAB, rabbit/mouse-general, two-step, GK500710, Gene Tech, Shanghai, China) according to the manufacturer’s proto- col. Images were acquired by polarized light microscopy.

**Enzyme-Linked Immunosorbent Assay (ELISA)**

Conditioned media (CM) were collected from LICCF and HuCCT1 cells, and the concentrations of CCL20 and CXCL5 were quantified using an ELISA kit (ELISA LAB, Wuhan, CHN) following the manufacturer's protocol. Each sample was analyzed in quintuplicate to ensure reproducibility. Optical density (OD) values were measured at a wavelength of 450 nm using a microplate reader, and cytokine concentrations were extrapolated from a standard curve. To account for variations in cell density, the results were normalized to the cell number.

**Preparation of Culture Medium**
Normal CM: LAMs were seeded in 6-well plates (approximately 5×10^5 cells per well) and cultured with complete medium (RPMI-1640 + 10% FBS) for 48 hours. L-methionine sulfoximine (MSO, 5 mM) or V-9302 (concentration: 10 μM) was added as required by the experimental conditions. The supernatant was collected, centrifuged (1000×g, 5 minutes) to remove cell debris, filtered through a 0.22 μm membrane, and stored at 4°C.

**Glutamine (Gln) detection**

Glutamine quantification was performed using the Glutamine Microplate Assay Kit (AS0160, SAB Biotech), following the manufacturer's protocol. Briefly, LICCF and HuCCT1 cells were cultured in glutamine-free RPMI 1640 medium for 24 hours. Subsequently, cells were treated with either V-9302 or a vehicle control. Post-treatment, cells were harvested and lysed via sonication. The resulting lysates were subjected to glutamine measurement, with data normalized to cell number to ensure accuracy.

**Dual-Luciferase reporter assay**

The ICC cell suspension was seeded into a 96-well plate at an appropriate density of 1.0 × 10^4 cells. The binding sites of SPINK1 on the CCL20 promoter were predicted using the Human Transcription Factor Database (HumanTFDB, <http://bioinfo.life.hust.edu.cn/HumanTFDB/>). The interaction between SPINK1 and the CCL20 promoter was investigated using a dual-luciferase reporter assay. The CCL20 promoter fragment was cloned into the pGL3-Basic vector. SPINK1 was then cloned into the pcDNA3.1-3xFlag vector (Hanbio, China), or SPINK1 was silenced. Firefly luciferase plasmid CCL20 or pGL3-Basic (NC) was co-transfected with the SPINK1 eukaryotic expression vector (containing pcDNA3.1-3xFlag) or the SPINK1 silencing vector. After 48 hours, cell lysates were collected, and firefly luciferase activity was measured using the Dual-Luciferase Reporter Assay Kit (HB-DLR-100, Hanbio, China).
